# Translational Algorithms for Technological Dietary Quality Assessment Integrating Nutrimetabolic Data with Machine Learning Methods

**DOI:** 10.3390/nu16223817

**Published:** 2024-11-07

**Authors:** Víctor de la O, Edwin Fernández-Cruz, Pilar Matía Matin, Angélica Larrad-Sainz, José Luis Espadas Gil, Ana Barabash, Cristina M. Fernández-Díaz, Alfonso L. Calle-Pascual, Miguel A. Rubio-Herrera, J. Alfredo Martínez

**Affiliations:** 1 Cardiometabolic Nutrition Group, Precision Nutrition Program, Research Institute on Food and Health Sciences IMDEA Food, Consejo Superior de Investigaciones Científicas-Universidad Autónoma de Madrid (CSIC-UAM), 28049 Madrid, Spain; edwin.fernandez@alimentacion.imdea.org (E.F.-C.); jalfredo.martinez@imdea.org (J.A.M.); 2 Faculty of Health Sciences, International University of La Rioja (UNIR), 26004 Logroño, Spain; 3 Endocrinology and Nutrition Department, Instituto de Investigación Sanitaria del Hospital Clínico San Carlos, Hospital Clínico San Carlos (IdISSC), 28040 Madrid, Spain; pilar.matia@gmail.com (P.M.M.); a.larrad47@gmail.com (A.L.-S.); ana.barabash@gmail.com (A.B.); alfonsoluis.calle@salud.madrid.org (A.L.C.-P.); marubioh@gmail.com (M.A.R.-H.); 4 Department of Medicine II, Faculty of Medicine, Complutense University of Madrid, 28040 Madrid, Spain; 5 Centro de Investigación Biomédica en Red de Diabetes y Enfermedades Metabólicas Asociadas (CIBERDEM), 28029 Madrid, Spain; 6 GENYAL Platform on Nutrition and Health, Research Institute on Food and Health Sciences IMDEA Food, Consejo Superior de Investigaciones Científicas-Universidad Autónoma de Madrid (CSIC-UAM), 28049 Madrid, Spain; cristinamaria.fernandez@imdea.org; 7 Centre of Medicine and Endocrinology, University of Valladolid, 47002 Valladolid, Spain

**Keywords:** precision nutrition, clinical biomarkers, nutritional evaluation, dietary assessment, machine learning, computational algorithm

## Abstract

Recent advances in machine learning technologies and omics methodologies are revolutionizing dietary assessment by integrating phenotypical, clinical, and metabolic biomarkers, which are crucial for personalized precision nutrition. This investigation aims to evaluate the feasibility and efficacy of artificial intelligence tools, particularly machine learning (ML) methods, in analyzing these biomarkers to characterize food and nutrient intake and to predict dietary patterns. Methods: We analyzed data from 138 subjects from the European Dietary Deal project through comprehensive examinations, lifestyle questionnaires, and fasting blood samples. Clustering was based on 72 h dietary recall, considering sex, age, and BMI. Exploratory factor analysis (EFA) assigned nomenclature to clusters based on food consumption patterns and nutritional indices from food frequency questionnaires. Elastic net regression identified biomarkers linked to these patterns, helping construct algorithms. Results: Clustering and EFA identified two dietary patterns linked to biochemical markers, distinguishing pro-Mediterranean (pro-MP) and pro-Western (pro-WP) patterns. Analysis revealed differences between pro-MP and pro-WP clusters, such as vegetables, pulses, cereals, drinks, meats, dairy, fish, and sweets. Markers related to lipid metabolism, liver function, blood coagulation, and metabolic factors were pivotal in discriminating clusters. Three computational algorithms were created to predict the probabilities of being classified into the pro-WP pattern. The first is the main algorithm, followed by a supervised algorithm, which is a simplified version of the main model that focuses on clinically feasible biochemical parameters and practical scientific criteria, demonstrating good predictive capabilities (ROC curve = 0.91, precision–recall curve = 0.80). Lastly, a reduced biochemical-based algorithm is presented, derived from the supervised algorithm. Conclusions: This study highlights the potential of biochemical markers in predicting nutritional patterns and the development of algorithms for classifying dietary clusters, advancing dietary intake assessment technologies.

## 1. Introduction

The fields of medicine and nutrition have made significant strides in biomarker development, driven by technological innovations linking health status, food intake, and metabolic well-being [1]. Among the most promising tools for practical application, metabolic biomarkers have become essential tools for measuring compounds related to biological or nutritional processes, playing a key role in improving medical diagnosis and personalized interventions [2]. Refining predictive and prognostic biomarkers is crucial for advancing personalized nutrition, offering quantitative tools to assess dietary intake, in line with advancements in dietary assessment technologies [3,4].

In human intervention trials, food and diet data commonly serve as key factors for understanding health outcomes. Indeed, the typical dietary pattern plays a vital role in the progression of chronic diseases and the preservation of an individual’s health [5]. Current dietary assessment methods, such as food frequency questionnaires (FFQs) and 24 h recalls, largely rely on self-reported data, leading to potential biases like recall bias, social desirability bias, and the misunderstanding of portion sizes. These methods largely depend on subjective interpretations and established food composition tables, resulting in data that may not be completely reliable, often being based on estimates rather than precise measurements. Specifically, self-reported dietary questionnaires can introduce various biases, such as recall bias, social desirability bias, and the misunderstanding of portion sizes, as well as pitfalls associated with food consumption table inaccuracies. These biases can lead to inaccuracies in the reported dietary intake, producing information that may not reflect true consumption patterns [6]. These limitations focus on a crucial gap in the current research on dietary intake assessment, as traditional methods may not provide the necessary accuracy for personalized nutrition. Furthermore, while biomarkers of food intake (BFIs) offer a promising alternative for more objective assessments, the existing BFI profiles are not yet able to precisely characterize specific dietary patterns, limiting their practical use in both clinical and research settings. This scenario represents a crucial gap in the field of precision nutrition [7].

The development of BFIs occurs through metabolomic studies carried out in samples of different biological fluids such as blood, plasma, and urine, which are expected to provide tailored information of dietary consumption and nutrient intake [8,9]. The characterization of BFIs underlies metabolomic studies conducted across diverse populations [10]. Explorations into metabolites within the realm of food have given rise to the term nutrimetabolomics, an emerging scientific discipline anticipated to play a crucial role in unraveling the complex interplay between diet and health [11]. The identification of BFIs allows for progress in the field of precision nutrition, since the interindividual variability that occurs in the analysis of biological fluids must be interpolated [1]. Therefore, it is a very useful tool for precision nutrition, an approach to diet and food assessment based on phenotypical/genotypical, dietary, metagenomic, and metabolic aspects in order to offer unique dietary recommendations for each individual [12].

The development of artificial intelligence (AI) tools, particularly the application of machine learning (ML) algorithms, has unexpected potential for identifying dietary patterns and predicting diseases [13]. The implementation of the study of BFIs from biological samples of urine and blood could represent a turning point for the characterization of dietary intake in different populations, allowing for a much more sensitive categorization for the recommendation of specific dietary patterns [14]. While food consumption is linked to dietary patterns that impact health, current BFI profiles are still limited in accurately pinpointing specific dietary patterns [15]. In this sense, AI, particularly ML, offers a pathway by processing complex biostatistical and bioinformatic data, helping to uncover patterns within BFIs that were previously undetectable. This approach could refine precision nutrition by analyzing phenotypic and metabolic data, enabling a better interpretation of dietary habits and health outcomes [16]. Translational bioinformatics, supported by AI, allows for the design of predictive models that improve our understanding of the relationship between diet and disease, offering new avenues and facilitating targeted dietary interventions [17,18].

In recent years, AI and ML algorithms have shown great potential in dietary research, offering new ways to analyze large-scale phenotypic, metabolic, and biochemical biomarker data for individualized nutrition. However, the use of AI also raises important ethical considerations, including the risk of biased data representation and potential privacy concerns [19]. These challenges must be carefully addressed to ensure that AI tools are used responsibly in nutrition research. Despite these issues, AI-driven tools remain promising for advancing biomarker research and improving dietary pattern assessment across diverse populations [20].

In this sense, the primary aims of the Dietary Deal project (Ref: AC21_2/00038), which is a European study focusing on multidisciplinary open-source dietary research, were to devise an assessment tool with ML capabilities for automatic classification for the harmonization of dietary pattern data across Europe and to advance ongoing biomarker research in nutritional status and health. This investigation evaluated the feasibility and effectiveness of utilizing bioinformatics tools, particularly ML algorithms, to analyze phenotypical, metabolic, and biochemical biomarkers, thereby enabling the characterization of food and nutrient consumption as well as the prediction of dietary patterns based on emerging technological methods.

## 2. Materials and Methods

### 2.1. Dietary Deal Substudy Procedure

This translational ancillary substudy from Dietary Deal was performed to compare two dietary assessment methods, the 72 h dietary recall (short-term) and FFQ (long-term) tools, with traditional measurements of food intake and to validate the relationships between the dietary intake data and metabolic biomarkers in the field of precision nutrition related to food consumption. Between November 2022 and June 2023, a total of 150 participants were enrolled in this study in the Hospital Clínico San Carlos in Madrid. The retention rate among participants was 92%.

The volunteers were recruited from the endocrinology/nutrition department (healthy members of hospital staff, patients without short bowel syndrome or changes in the gastrointestinal tract, and subjects not taking oral supplements or vitamins). The demographic characteristics of the participants included the following: 90.67% Caucasian, 30.43% males, and 69.57% females, ranging 18–79 years (P25-P75: 30–55 years). Recruitment was carried out by specialist medical doctors and trained health staff. Sample size estimation was calculated using the formula for correlation studies [21]: (n = ([z_1−α_ + z_1−β_]^2^/z_0_^2^) + 3), where α is the two-side significance (0.05), β is the predefined statistical power in 80% (0.2), and z0 is the Fisher’s z transformation of the population correlation coefficient that can be taken from tables. Assuming an expected moderate correlation (r = 0.4 between BFIs and diet), we obtained a minimum of 47 participants.

**Figure 1:**
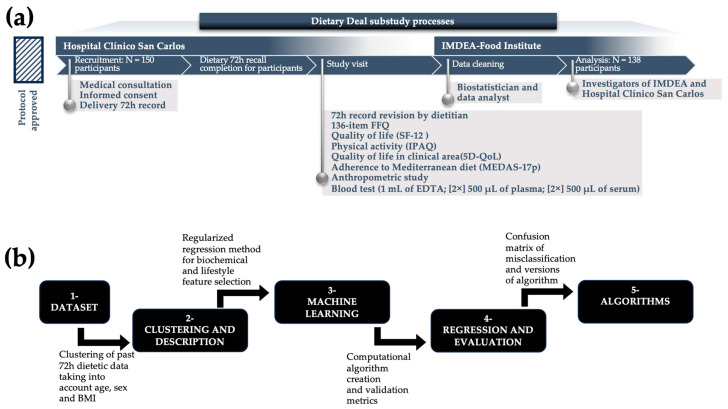
Graphical abstract: (**a**) flowchart; (**b**) graphical representation of analysis sequence. Abbreviations: BMI, body mass index, FFQ, food frequency questionnaire.

In the Dietary Deal project, we collected data in person through a team of specialists, including doctors, registered dietitians, lab technicians, and research staff (see Figure 1). Initially, medical doctors assessed the eligibility of patients to participate in this study. Once the patients provided informed consent—both written, as a signed informed consent form, and verbal—the staff specialists collected the step patients’ clinical data and medical history during a thorough examination. After this, patients met with a nutritionist who explained how to fill out dietary questionnaires and collect urine and blood samples. Within 5 to 7 days, patients were scheduled for an 8 h fasting period at the same hospital for blood sample collection. Following these procedures, a specialized nutritionist reviewed the patients’ dietary records from the past 72 h and helped complete dietary, quality of life, and physical activity questionnaires, as well as calculating their body mass index (BMI). The Research Ethics Committee of Hospital Clínico San Carlos approved this study (approval number: CEI 22/363-E, 7 June 2022).

### 2.2. Biochemical Markers

Biochemical data comprised those from the standard profiling routinely performed at Hospital Clínico San Carlos, encompassing a total of 80 parameters categorized into the following groups: hematology (21 variables); coagulation (6 variables); metabolites (13 variables); enzymes (4 variables); ions (6 variables); inflammation and sepsis (2 variables); trace elements (3 variables); and hormones, vitamins, and metabolism (22 variables). Selenium, the vitamin A/retinol binding protein ratio, and vitamins B1 and B6 were excluded due to a lack of >20% of missing data. To address missing values, a robust imputation strategy was applied, replacing those parameters exhibiting 0–20% missingness with their respective medians, ensuring a comprehensive and representative dataset for the subsequent analyses.

### 2.3. Dietary Intake Assessment

The selection of dietary assessment tools and statistical methods in this study was based on their ability to comprehensively capture both short- and long-term dietary intake and their validation in a similar community based in the Spanish population. Food intake was assessed using different questionnaires: a 136-item semi-quantitative FFQ, 72 h recall questionnaire, and Mediterranean diet adherence questionnaire. Notably, the information obtained from the 136-item FFQ was used to establish participants’ long-term dietary patterns, while the 72 h recall questionnaire provided a closer look at their dietary intake in the three days leading up to data collection. The 136-item semi-quantitative FFQ considered the consumption frequency over the past year to evaluate the long-term dietary pattern. This FFQ was validated in Spain and subsequently re-evaluated [22,23]. Food consumption patterns were asked about in each item of the FFQ with a single frequency response ranging with the following nine possible response categories: never/seldom, 1–3 servings/month, 1 serving/week, 2–4 servings/week, 5–6 servings/week, 1 serving/day, 2–3 servings/day, 4–6 servings/day, and >6 servings/day. Standard portion sizes were also specified. The daily consumption of every food item was estimated by multiplying the typical portion size frequency of consumption using an ad hoc computer program specifically developed for this aim [24,25]. Short-term food and nutrient intakes were assessed using a 72 h diet recall considering the preceding intakes 3 days before blood extraction. In a face-to-face interview, well-trained dietitians studied all food consumed in the preceding 72 h, including the methods of food preparation, foods eaten outside the home, nutrition supplements, and beverages. Completed food records, energy, and nutrient intake were calculated using the DIAL computerized nutrient analysis program with accepted food composition tables [26]. Also, healthy diet was assessed using the 14- and 17-item Mediterranean Diet Adherence Screener (MEDAS-14 and MEDAS-17) previously validated in a Spanish cohort [27,28], along with their ability to assess adherence to healthy dietary patterns relevant to cardiovascular health. The Alternate Healthy Eating Index 2010 (AHEI-2010, [29]) was chosen for its comprehensive approach to capturing dietary components linked to chronic disease prevention. Lastly, diet quality was assessed using three previously published diet quality indices from FFQ information: Dietary Approaches to Stop Hypertension (DASH, [30]), Pro-vegetarian Dietary Pattern (PVG, [31]), and Mediterranean diet score (MDS, [32]). The first of these quality indices is relevant to blood pressure regulation (particularly in populations at risk for hypertension); the second captures the extent of adherence to plant-based diets (an important dietary shift associated with lower cardiovascular risk); and the third is a well-established measure of Mediterranean dietary adherence, reinforcing the consistency of dietary pattern assessments across different indices.

### 2.4. Potential Predictors

This trial encompassed potential predictors for food and nutrient intake, including demographic, lifestyle, and health-related data. Participants’ demographic information consisted of variables such as sex (male/female), age (years), and self-reported ethnicity (Caucasian, South American). Tobacco use (non-smokers, former, current) and prevalence (yes/no) and family medical history (yes/no) were examined through clinical information, including a history of cardiovascular disease (CVD), dyslipidemia, high blood pressure (HBP), type 2 diabetes (T2D), obesity, depression, and insomnia, among other prevalent diseases. The number of hours per week of physical activity was determined by multiplying the number of days per week by the hours per day and dividing the result by 60. This estimation was applied separately for intense, moderate, and light physical activity, based on the responses to the relevant questions in the International Physical Activity Questionnaire (IPAQ, [33]). Responses to the self-reported quality of life (QoL) using the 12-item Short-Form questionnaire (SF-12) were collected [34].

### 2.5. Statistical Analysis

Baseline characteristics were described as the mean ± standard deviation (SD) or median ± interquartile range (IQR) for continuous variables, and the percent for frequencies was used to describe characteristics adjusted for age and sex using the inverse probability weighting (IPW) method [35]. A parametric or nonparametric contrast of hypothesis was applied according to the normality of each variable using the Shapiro–Wilk test. A summary of the statistical methodology is provided in Figure 1. The process started with the clustering of participants based on specific criteria, such as dietary intake, age, and sex, which form the basis of the dataset. Following this, descriptive statistical analyses were conducted alongside clustering techniques to categorize the data effectively. The workflow progressed into machine learning, where advanced computational algorithms were employed for data analysis, model training, and validation aimed at predicting dietary intake. Finally, regression analyses were performed to evaluate the relationships between dietary variables and health outcomes, culminating in the development of algorithms based on these evaluations and ROC validations. Statistical analyses encompassed comparisons of the means or medians across categories, employing Student’s *t*-test for normally distributed data and the Mann–Whitney test for nonparametric datasets.

To construct the similarity-based clusters, the following methodological steps were undertaken: First, we implemented cluster analysis using data obtained from the 72 h dietary recall, considering sex, age, and BMI. Second, we assigned names to each cluster through exploratory factor analysis (EFA), based on food consumption patterns and nutritional scores derived from the FFQ, which reflects participants’ long-term dietary patterns. For the first step, we explored three clustering algorithms—Ward hierarchical clustering, non-hierarchical k-means, and partitioning around medoids—alongside four validation metrics, including bootstrap silhouette widths [36], the Dunn index [37], the connectivity of cluster [38] statistics, and the visual bootstrap elbow method. The clValid package in R (version 0.7) [39] facilitated internal cluster validation, helping us to determine the optimal number of clusters. We applied hierarchical clustering to uncover hidden structures within the datasets. Initially, we created a dissimilarity matrix using the dist function in R (stats package version 4.3.0), applied to the dataset. Next, we constructed a hierarchical clustering model using the hclust function (stats package version 4.3.0) with the Ward.D2 method [40]. The Ward.D2 method, known for minimizing variance, was chosen to identify meaningful clusters in datasets with diverse sizes and configurations. The resulting hierarchical clustering model reflects inter-point relationships, allowing us to identify distinct clusters [40]. The resulting hierarchical clustering model summarizes inter-point relationships, facilitating the identification of distinct clusters within the dataset. For the second step, we assigned names to each cluster through dietary EFA using the most representative FFQ food groups measured in grams per day (vegetables, fruit, nuts, pulses, fish, olive oil, other fats excluding olive oil, whole grains, refined grains, whole dairy products, red meat, lean meat, pastries and cookies, total energy intake from ultra-processed foods, and total dairy products) to understand long-term dietary patterns. We also considered the MDS, AHEI-2010, MEDAS-14, MEDAS-17, and PVG scores in this analysis. Loadings from the EFA helped us assess adherence to each factor within different groups and identify enduring dietary characteristics associated with each cluster.

To develop the translational computational algorithm, the following methodological steps were undertaken: First, we selected key biochemical and lifestyle variables associated with clusters using elastic net regularization. Second, after optimizing the model, we applied Logistic Regression to generate coefficients for each variable associated with clusters, forming the algorithm. Lastly, a random forest model was used to further assess variable importance, ensuring the algorithm’s predictive accuracy and practical relevance as well as the contribution of independent variables. For the first step, we first identified the most distinct biochemical and lifestyle-related variables using Logistic Regression with elastic net regularization and 5-fold cross-validation using the cv.glmnet function from the glmnet package in R (version 4.1-8) [41]. ML methods were applied using an 80% training and testing set, reserving the remaining 20% for validation, which was split during model training. We used various metrics to find the optimal alpha (α) and lambda (λ) values that minimized the root mean squared error. After model training, we used the optimized alpha hyperparameter to identify the biochemical and lifestyle-related variables linked to each cluster. The training process included hyperparameter tuning via grid search to ensure optimal model performance, while cross-validation helped prevent overfitting. The main challenge encountered was the variability in performance across different folds, requiring adjustments in feature selection and model tuning to improve generalizability. We then conducted Logistic Regression with the selected variables to derive coefficients (β) for each variable, forming the computational algorithm. We used the Logistic Regression equation for probability calculations, assigning a weight to each variable based on the β from the model. Furthermore, we deployed a random forest model to assess the significance of each variable, involving 500 bootstrap iterations for a robust evaluation of variable importance. We systematically applied several performance metrics to provide a nuanced understanding of the model’s predictive capabilities, including the area under the curve (AUC), precision–recall AUC (PR AUC), true positive (TP, %), true negative (TN, %), false positive (FP, %), false negative (FN, %), correctly classified instances (%), and the percentages of positive and negative predicted outcomes. Additionally, we created two simplified versions of the algorithm (a supervised computational algorithm and a biochemical computational algorithm) based on scientific and practical criteria. The selection process involved a thorough performance evaluation using the previously mentioned metrics, ensuring a balanced and refined algorithmic representation. This streamlined version aimed to maintain scientific rigor while enhancing practical applicability, emphasizing the careful consideration of various metrics in shaping the final algorithm.

Statistical analysis was performed using STATA (version 18.0, StataCorp., College Station, TX, USA) and RStudio (version R 4.3.0, 2022.12, PBC) software. A two-sided contrast of hypothesis with a *p*-value  <  0.20 was classified as a marginal trend of statistical significance, whereas differences were considered statistically significant at a *p*-value  <  0.05.

## 3. Results

The results of the participants’ dietary patterns are presented below and were obtained by applying clustering analysis and nomenclature assignment based on a 72 h dietary recall. Additionally, we described the sociodemographic differences, short-term dietary behaviors, and biochemical markers linked to each dietary cluster. Finally, the results of computational algorithms designed to predict classifications based on the identified dietary patterns are also presented.

### 3.1. Clustering and Nomenclature Assignment

The cluster analysis, based on the 72 h dietary recall data, showed that Ward hierarchical clustering was the best method for grouping participants. The analysis identified two clusters (k = 2) as the optimal number (Figure S1). In the first cluster (C1), there were 86 participants, while the second cluster (C2) had 52 participants (Figure S1). We examined basic sociodemographic differences between males and females (Table 1).

**Figure 2:**
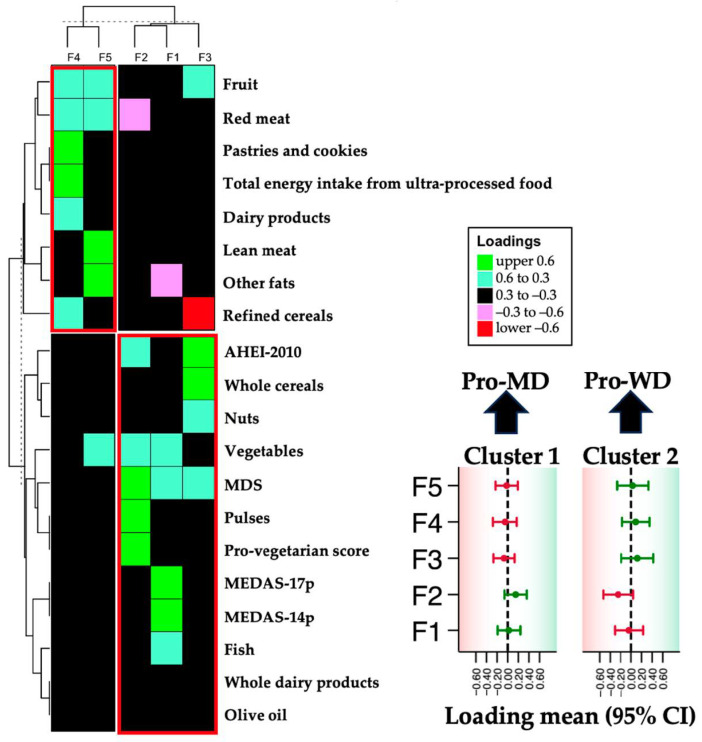
Visualization of factor loadings after exploratory factor analysis to assign name clustering. Abbreviations: MEDAS, Mediterranean Diet Adherence Screener; pro-MP, pro-Mediterranean pattern; pro-WP, pro-Western pattern.

The results indicated that males had a higher body mass index (BMI) (*p* = 0.024), fat mass percentage (*p* < 0.001), prevalence of type 2 diabetes (T2D) (*p* = 0.004), and glucose levels (*p* = 0.016) compared to females. Males also had a higher rate of smoking (*p* = 0.037). Next, we named each cluster using exploratory factor analysis (EFA) based on 15 food groups from the food frequency questionnaire (FFQ) and five dietary quality indices, which reflect long-term eating patterns. These groups and quality indices were included in the analyses because they were the specific categories available in the dataset derived from the food frequency questionnaire (FFQ). This selection allowed for a thorough evaluation of the dietary patterns of participants based on the information at hand. The EFA revealed five main factors that describe the participants’ dietary habits. Each factor represents a specific dietary pattern, making it easier to classify diets and understand their health implications. The visualization of the factor loadings (Figure 2) shows a clear distinction between foods linked to each factor, explaining 60% of the variation (R^2^ = 60). After applying a varimax rotation to the factor loadings, we created a heatmap that showed two main groups: Batch 1 (Factor 1, Factor 2, Factor 3) included factors related to a pro-Mediterranean pattern (pro-MP), featuring whole grains, nuts, vegetables, legumes, fish, and Mediterranean diet scores. Batch 2 (Factor 4, Factor 5) was linked to a pro-Western pattern (pro-WP) characterized by red meat, pastries, ultra-processed foods, refined grains, and fats other than olive oil. Overall, the EFA results indicate that the diets of the study participants can be classified into distinct dietary profiles.

### 3.2. Descriptive Analysis of Short-Term 72 h Dietary Results by Clusters

A descriptive analysis of the short-term 72 h dietary results with significant (*p* < 0.05) and marginal trends (*p* < 0.20) is presented to highlight significant distinctions in plant-based dietary preferences between the pro-MP and pro-WP clusters (Table 2). The short-term dietary data from the 72 h dietary recall suggest potential differences in these dietary inclinations.

Regarding tubers, the pro-MP cluster exhibits a greater inclination for acceptance, registering only 2.3% non-consumers. The median consumption of root tubers stands at 61.2 g/d, with a range of variability between 32.8 g/d and 87.7 g/d. Conversely, the pro-WP fraction manifests 19.2% non-consumers, a median of 56.6 g/d, and a narrower variability (IQR: 23.6–80), with a nearly significant divergence (*p* = 0.052). Regarding canned legumes, the pro-MP fraction exhibits a substantial proportion of non-consumers (95.3%), while the pro-WP fraction shows 88.5%, though lacking statistical significance. For the median consumption of dried legumes, the pro-MP cluster records 58.1% non-consumers, while the pro-WP cluster logs 75%, evincing a marginal trend toward significance (*p* = 0.091). Within the category of cereals, the pro-MP cluster evinces high acceptance (1.2% non-consumers), compared with pro-WP at 1.9% non-consumers, featuring variability in preferences. Notably, in breakfast cereals, the pro-MP cluster reflects a considerable percentage of non-consumers (82.6%), whereas the pro-WP cluster exhibits 63.5%, indicating a significant disparity (*p* = 0.01). Nevertheless, for grain flour, distinctions between groups are non-significant, albeit the pro-MP cluster displays more non-consumers, with diminished consumption among the participants.

Concerning beverages, the pro-WP cluster showcased elevated overall consumption (*p* < 0.001), with marked disparities in alcoholic beverages (*p* = 0.023), indicating higher consumption in the pro-MP cluster, and non-alcoholic beverages (*p* < 0.001), denoting increased consumption in the pro-WP cluster. Commercial juices, conversely, witnessed a significantly augmented consumption in the pro-WP cluster (*p* = 0.022). The data revealed a non-exacerbated distinction in protein dietary inclinations between the pro-MP and pro-WP clusters (Table 2). Examining meat consumption, specifically sausages, no significant differences were discerned between the groups, although the pro-WP cluster displayed a proclivity for greater consumption. In the context of dairy products, the pro-MP cluster manifests a lower consumption of cheese compared to the pro-WP cluster (median of 30 vs. 35.5 g/d, *p* = 0.044). Lastly, with respect to fish, the pro-MP cluster recorded 14% non-consumers, while the pro-WP cluster registered 17.3%, devoid of significant disparities. Fatty fish consumption among the pro-MP cluster revealed a notable proportion of non-consumers (79.1%), while smoked fish, within the pro-MP cluster, noted 86% non-consumers and the pro-WP cluster 96.2%, exhibiting a marginal inclination toward significance (*p* = 0.056). For more details of all descriptive analyses of the short-term 72 h dietary results by clusters, see Table S1.

### 3.3. Descriptive Analysis of Biochemical Markers by Clusters

A descriptive analysis of the biochemical marker results with significant (*p* < 0.05) and marginal trends (*p* < 0.20) is presented to highlight significant distinctions between the pro-MP and pro-WP clusters (Table 3).

A noteworthy finding was the significant disparity in HDL cholesterol levels (*p* = 0.004). The pro-MP cluster demonstrated elevated HDL cholesterol levels in comparison to the pro-WP cluster. In the context of renal function, a significant difference was noted in the glomerular filtration rate estimated by collaboration in the epidemiology of chronic kidney disease (CKD-EPI) (*p* = 0.041), despite comparable median values. Additionally, the Tocopherol/cholesterol ratio exhibited a significant difference (*p* = 0.031), showing higher levels in the pro-WD cluster (7.6 vs. 7.4).

Metabolic markers showing a marginal trend included zinc (*p* = 0.051), white blood cells (*p* = 0.140), lymphocytes (*p* = 0.096), monocytes (*p* = 0.090), basophils (*p* = 0.080), total cholesterol (*p* = 0.128), ceruloplasmin (*p* = 0.138), and vitamin C (*p* = 0.056). The pro-WP cluster exhibited a trend towards higher zinc levels. Furthermore, the pro-MP group showed diminished levels of white blood cells. Although no significant differences in lymphocyte count were found, a trend towards elevated numbers was noted in the pro-WP group. Concerning total cholesterol, the pro-MP group demonstrated a trend towards higher levels compared to the pro-WP group, and the pro-MP group also exhibited a tendency towards higher interquartile range values for vitamin C concentration. For more details of all descriptive analyses of biochemical markers, see Table S2.

### 3.4. Computational Algorithm

#### 3.4.1. Main Algorithm

**Figure 3:**
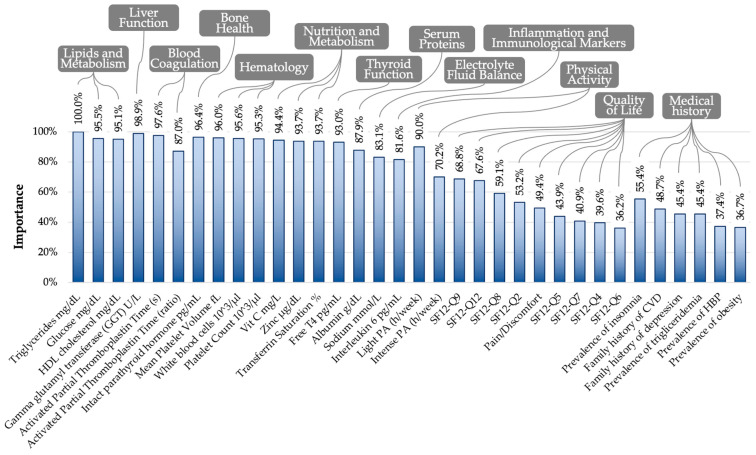
Estimation of model variable importance through utilization of random forest model of main model. Abbreviations: HBP, high blood pressure; CVD, cardiovascular disease; HDL, high-density lipoprotein; PA, physical activity; SF-12, 12-item Short-Form questionnaire.

For the development of the computational algorithm predicting classification into the pro-WP cluster, Logistic Regression with elastic net regularization was employed, utilizing 5-fold cross-validation. After model training, it was observed that the optimal alpha value minimizing the root mean squared error was 0.2 (Figure S2). The number of BFIs selected according to the chosen alpha was 34 (Figure S2). The identified BFIs encompass a range of physiological and lifestyle factors, including platelet count, mean platelet volume, white blood cells, activated partial thromboplastin time, activated partial thromboplastin time, glucose, HDL cholesterol, albumin, triglycerides, gamma glutamyl transferase, sodium, interleukin-6, zinc, free T4, vitamin C, transferrin saturation, intact parathyroid hormone, intense physical activity, light physical activity, family history of CVD, depression, hypertriglyceridemia, HBP, obesity, insomnia, some questions of the quality of life questionnaire (Q2, Q4, Q5, Q6, Q7, Q8, Q9, Q12), and pain/discomfort. Subsequently, Logistic Regression was applied to obtain β coefficients for each of the 34 selected BFIs, assigning weights based on the response provided for each patient (Table S3). The model explained a substantial portion of the variance (R^2^ = 81.9%). We calculated the importance of each biomarker in the main computational algorithm through the application of a random forest classification model with 500 iterations, providing the percentage importance of each biomarker considering the clusters as the dependent variable (Figure 3). It was observed that biomarkers related to lipid and metabolism, liver function, blood coagulation, bone health, hematology, nutrition and metabolism, thyroid function, and light physical activity were the most critical (score importance > 90%). BFIs with less weight in the model were found to be those related to intense physical activity, quality of life, and medical history (score importance between 36.2% and 70.2%).

The model’s performance metrics for classification demonstrated a receiver operating characteristic (ROC) curve area of 0.99; a PR AUC of 0.60; acceptable sensitivity vs. specificity; and a prediction of TP and TN of 96.15%, FP of 2.33%, and FN of 3.85% (Table S4).

#### 3.4.2. Supervised and Supervised-Only Biochemical Computational Algorithms

**Figure 4:**
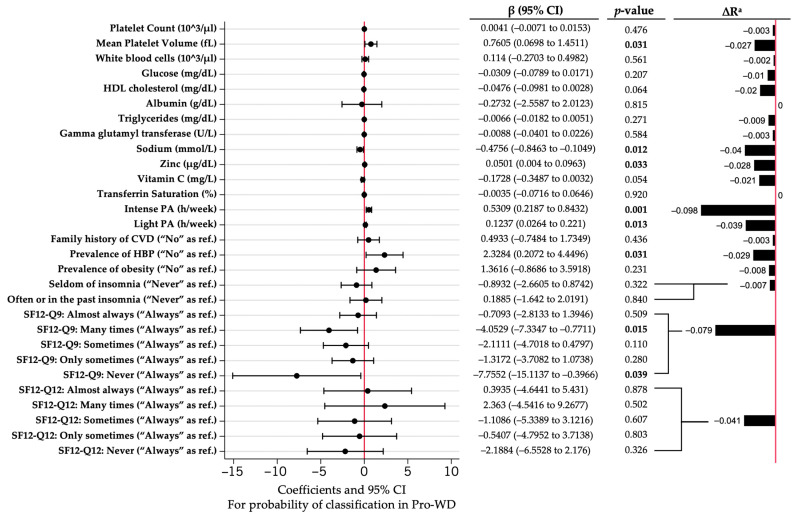
Logistic Regression coefficients for probability of classification in pro-WD using supervised computational algorithm. Abbreviations: PA, physical activity; C, cluster; CI, confidence interval; CVD, cardiovascular disease; HBP, high blood pressure; HDL, high-density lipoprotein; SF-12, 12-item Short-Form questionnaire. ^a^ ∆R represents R change after subtracting each variable from full model. Red line represents the reference null value (β = 0) for the model, while the bold highlights mean that there is statistical significance.

A simplified version of the main computational algorithm was devised based on both scientific and practical criteria (Figure 4). This supervised computational algorithm was selected for biochemical parameters that are more practical in clinical settings and involve fewer questions for response. The mean platelet volume, sodium, zinc, physical activity (both intense and moderate), the prevalence of hypertension, and SF12-Q9 exhibited significant associations in predicting the probability of classification within the pro-WD cluster using our supervised computational algorithm. The most influential contributions to the model were physical activity and SF12-Q9, which demonstrated the highest ∆R^2^ (Figure 4). Additionally, the Logistic Regression model for this new supervised algorithm accounted for a moderate to high fraction of the variance, with an R^2^ value of 44.3% (Figure 4).

**Figure 5:**
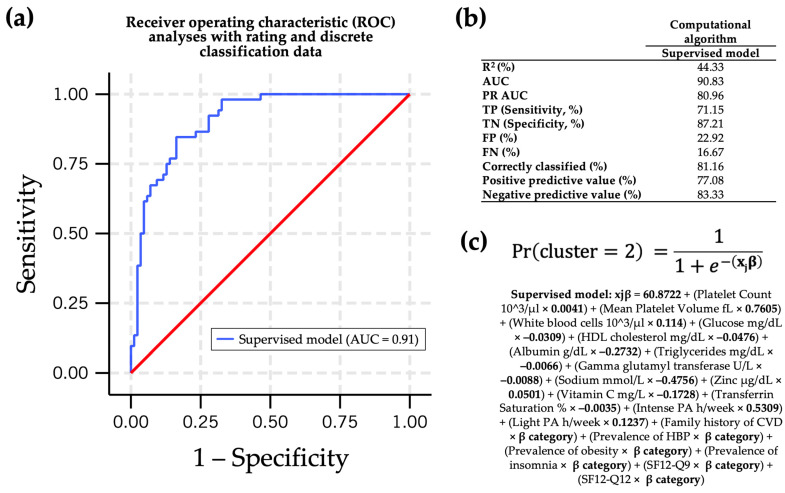
Evaluation metrics and computational algorithm. (**a**) Assessment of supervised model discriminatory performance between positive and negative classes via receiver operating characteristic (ROC) analyses. Red line represents the reference value (AUC = 0.5) for the ROC curve; (**b**) confusion matrix; (**c**) computational algorithm. Abbreviations: AUC, area under the curve; CVD, cardiovascular disease; FN, false negative; FP, false positive; HBP, high blood pressure; PA, physical activity; PR, precision–recall; TN, true negative; TP, true positive.

Analysis also shows an ROC curve area of 0.91; a PR AUC higher than the main computational algorithm = 0.80; sensitivity vs. specificity lower than the main computational algorithm but moderately acceptable; and a prediction of TP of 71.15%, TN of 87.21%, FP of 16.67%, and FN of 22.92%, metrics lower than those of the main computational algorithm (Figure 5). No substantial improvements were seen, although a good–moderate prediction was observed according to the ROC curve, when we further reduced the number of variables in the supervised model, leaving only the biochemical one (named as supervised-only biochemistry model in Table S3). For the construction of computational algorithms, coefficients from the Logistic Regression model equation were considered (Table S5). The overall performance of the supervised computational algorithm appears to excel in most assessed aspects, underscoring its efficacy in the specific task at hand.

## 4. Discussion

In light of the limitations associated with current dietary assessment methodologies, such as inaccuracies in food composition databases, subjective recall, and the reliance on standardized nutritional guidelines that may not fully capture individual dietary patterns and their implications for health, there arises a pressing need for more advanced and precise tools in nutritional science. In this prospective study, we implemented an ML methodology to improve the predictions of algorithms. First, we developed a clustering analysis using data obtained from the 72 h dietary recall, taking into account the sex, age, and BMI of participants. Second, for the assignment of nomenclature to each cluster, we used an EFA, grounded in food consumption patterns and nutritional indices derived from the FFQ (knowledge of the long-term dietary pattern of participants). Through cluster and exploratory factor analysis, we identified two dietary patterns, pro-MP and pro-WP. Third, we applied a training–testing elastic net regression analysis to obtain the best biomarker functional indicators associated with the patterns defined. This model derived β coefficients for 34 BFIs. Lastly, three computational algorithms were created to predict the probabilities of being classified into the pro-Western pattern: (1) the main algorithm; (2) the supervised algorithm, a simplified version of the main model, focusing on clinically feasible biochemical parameters and scientific and practical criteria, with good to moderate strong predictive capabilities (ROC curve = 0.91, precision–recall curve = 0.80); and (3) the only biochemical algorithm derived from the supervised algorithm.

From a population health perspective, these findings underscore the importance of promoting adherence to healthier dietary patterns, such as the Mediterranean diet, particularly in regions facing increasing rates of diet-related chronic diseases. Our machine learning (ML) model’s ability to differentiate these patterns using biomarkers offers a more objective understanding of how diet impacts health beyond self-reported intake, with significant implications for personalized nutrition and public health. Moreover, biomarker-driven interventions could lead to more effective strategies for disease prevention and health promotion at the population level.

The use of biomarkers to predict dietary intake provides deeper insight into how individual biological responses influence dietary choices and nutritional needs. This has practical relevance: identifying individuals at a higher risk of unhealthy dietary behaviors through biomarkers enables the development of personalized interventions tailored to their specific metabolic profiles. Furthermore, the capacity to detect even minor deviations from recommended dietary patterns allows for earlier interventions, potentially preventing the onset or progression of diet-related health conditions. Our study also highlights the need to shift from broad, traditional dietary guidelines to more individualized approaches that consider metabolic variability. This is especially crucial given the global rise in non-communicable diseases. Precision nutrition, as supported by our findings, could be instrumental in addressing in individual adverse community health challenges by offering adaptable solutions for diverse populations. Nevertheless, further research is required to refine these models and validate their effectiveness across different demographic groups, ensuring applicability to varied genetic, lifestyle, and cultural contexts. A pivotal aspect of precision nutrition lies in the accurate assessment of food intake and accompanying nutrients [42]. While traditional methods such as dietary questionnaires and food diaries have been widely used, they are inherently prone to biases and inaccuracies [43]. Herein lies the potential of nutrimetabolic approaches, which can take advanced technologies including web-based platforms, image-based methodologies, and wearable devices to provide a more precise and real-time assessment of dietary intake [44]. By incorporating BFIs into the nutritional assessment toolkit, nutritionists not only enhance the accuracy and specificity of dietary evaluations but also gain deeper insights into the complex relationships between diet and disease onset [45]. As we move towards a future where personalized nutrition becomes the cornerstone of healthcare interventions, the integration of BFIs offers a promising avenue for redefining dietary guidelines and optimizing health outcomes on an individualized basis [46,47].

The BFIs identified in this study, such as lipid profiles and metabolic indicators, have proven essential in differentiating pro-MP (C1) from pro-WP (C2) dietary patterns. Biologically, lipid profiles, particularly HDL cholesterol levels, are associated with cardiovascular protection [48]. Elevated HDL levels in the pro-MP group suggest a lower incidence of cardiovascular diseases and better metabolic health compared to the pro-WP group. Notably, the Mediterranean diet is linked to higher HDL levels than those in the Western diet, a trend also observed in our population, where women exhibited higher HDL levels than men. Moreover, triglyceride concentrations inversely affect HDL levels, and alcohol consumption plays a significant role in this relationship. In our cohort, women who reported lower alcohol intake had correspondingly higher HDL levels, observing the influence of lifestyle factors on lipid profiles. Furthermore, the pro-WP pattern was characterized by specific BFIs that provide insights into the potential health implications of this dietary pattern. The platelet count and mean platelet volume were significantly higher in the pro-WP group, indicating increased platelet activation and a potential pro-thrombotic state. A higher MPV is often associated with a higher risk of cardiovascular events, reflecting the pro-inflammatory nature of the pro-WP diet [49,50].

Our findings reveal a significant reduction in activated partial thromboplastin time in the pro-WP group. While the evidence is limited, some studies suggest that reduced activated partial thromboplastin time is indicative of an increased level of coagulation activity [51]. Vermylen et al. postulate that a shorter activated partial thromboplastin time may signal a hypercoagulable state, which is a known risk factor for thrombotic events, thereby highlighting potential adverse effects associated with the pro-WP diet [52]. Additionally, a genetic analysis by Weihong Tang et al. suggests a link between activated partial thromboplastin time and coronary artery disease, identifying specific genetic loci that correlate with coagulation factor deficiencies and elevated disease risk [51].

Elevated glucose levels, though marginally significant, in conjunction with significantly increased triglycerides, suggest a predisposition to metabolic syndrome in the pro-WP group [53]. Thus, Pramono et al. investigated the relationship between unhealthy food consumption and impaired glucose metabolism, discovering that the intake of sweet, grilled, and processed foods is associated with elevated fasting plasma glucose levels, indicating a connection between processed food consumption and glucose intolerance [54]. Furthermore, a meta-analysis evaluating diet quality and blood glucose levels across 15 cohorts found favorable associations between adherence to a healthy diet, as indicated by diet scores, and lower fasting glucose (β = −0.004, *p* < 0.0001) and insulin (β = −0.008, *p* < 0.0001) concentrations [55].

Recent research has implicated diet-related low-level metabolic acidosis in the pathogenesis of metabolic disorders, including metabolic syndrome, diabetes, and cardiovascular diseases [56], particularly highlighting the triggering effects of a Western dietary pattern [57]. Our findings are supported by a recent systematic review and meta-analysis that demonstrated that a higher dietary acid load is associated with increased triglyceride concentrations [58]. Furthermore, a review examining dietary patterns and triglyceride levels indicated that diets high in red meat, processed meat, refined grains, and potatoes are associated with elevated plasma triglyceride levels [59].

Additionally, the lower albumin levels and reduced gamma-glutamyl transferase in the pro-WP group may suggest liver marginal dysfunctions or poor protein status, potentially attributable to a diet high in processed foods and low in quality proteins [60,61]. Our results align with the findings of Lin et al., which indicate that adherence to a Western dietary pattern is associated with lower albumin levels [62]. Furthermore, several studies corroborate our observation of low serum albumin concentrations, highlighting significant relationships between inflammation markers associated with Western diets and albumin levels [63,64]. For example, data from the National Health and Nutrition Examination Surveys (1999–2004) showed that each 1 mg/dL increase in C-reactive protein independently correlated with a 1.02 odds ratio (*p* = 0.0003) for lower serum albumin levels [65]. Additionally, elevated levels of intercellular adhesion molecule-1, a vascular endothelial glycoprotein upregulated by inflammation, were independently linked to a 67% increased risk of sustained low serum albumin levels [66].

Several studies cited in a review have associated high-salt diets, such as the pro-WP diet [67], with chronic diseases including hypertension, kidney disease, and cardiovascular disease. For instance, Suckling et al. demonstrated that an increase in dietary salt intake, even from a single meal, rapidly raises plasma sodium concentration and blood pressure [68]. The pro-WP diet predominantly includes processed foods high in sodium. Although many studies have found a direct relationship between high sodium intake and health issues [69], it is possible that our study observed a “compensation effect”, where individuals with higher blood sodium levels may modify their diet to reduce the intake of high-sodium foods, thereby managing their health better.

Zinc is essential for immunometabolic functions [70], and studies such as that by Klein et al. [71] have found a higher proportion of individuals with zinc levels below the reference range in vegan and vegetarian groups, reinforcing the conclusion that Western diets generally exhibit higher plasma zinc concentrations compared to predominantly plant-based diets. Moreover, a meta-analysis indicated that serum zinc concentrations in vegetarians and vegans are lower than those in individuals following Western-based diets [72]. Western diets, which include highly bioavailable dietary sources of zinc from animal origins [73], facilitate adequate levels of both total and free zinc [73]. In contrast, plant-rich diets such as vegetarian and vegan diets often contain zinc absorption inhibitors like phytates [74], which bind to zinc and reduce its absorption in the intestine, resulting in lower plasma zinc levels [74].

Moreover, T4 plays a role in the normal functioning of various systems including the cardiovascular, metabolic, nervous, musculoskeletal, and reproductive systems [75]. Although limited studies have investigated the association between nutrition and thyroid function, some authors corroborate our finding of lower free T4 levels in the pro-WP group [76]. Noteworthy, Moslehi et al. identified that dietary patterns with high levels of intake of foods like pizza, soft drinks, processed meats, fast foods, pickled vegetables, breads, and soft drinks were associated with increased odds of subclinical hypothyroidism [76]. Additionally, two cross-sectional studies [77] using a predefined dietary index demonstrated a negative correlation between adherence to the Mediterranean diet and FT4 levels and a positive correlation between the dietary inflammatory index and total T4 levels [78].

These findings reinforce the detrimental effects of a pro-Western diet and the importance of a balanced intake of nutrients. The development of computational algorithms for predicting dietary pattern classification represents a significant advancement in clinical practice [79,80]. Biologically, these algorithms capture the complexity of nutrient–metabolism interactions [80]. The implementation of a Logistic Regression-based algorithm that achieves moderate accuracy (PR curve: 0.60) and a simplified version with similarly notable accuracy (PR curve: 0.80) demonstrates the potential of these tools to integrate multiple biomarker variables and predict dietary outcomes. These algorithms allow for the personalization of nutritional interventions based on individual profiles, thus optimizing biological benefits, such as improved cardiovascular and metabolic health and a reduced risk of chronic diseases [81]. This capacity for accurate dietary pattern prediction facilitates tailored dietary recommendations, maximizing positive health outcomes. In this context, the computational algorithms developed in this study have significant potential for real-world application in clinical settings. These models can be utilized in personalized nutrition counseling, enabling healthcare professionals to tailor dietary recommendations based on individual biomarker profiles and dietary patterns. For instance, dietitians can integrate these algorithms into their practice to identify patients at a higher risk of metabolic disorders or cardiovascular diseases, thus guiding targeted interventions.

Biologically, the precise identification of dietary patterns through biomarkers allows for a better understanding of the influence of the intake of different dietary components and physiological and metabolic processes [82]. The clear differentiation between the pro-MP and pro-WP dietary patterns and their respective impacts on health markers reveals how the impact of certain foods and nutrients enhances or impairs metabolic and cardiovascular functions. Conversely, the preference for commercial beverages and juices in the pro-WP group may relate to the higher intake of added sugars and preservatives, increasing the risk of insulin resistance and metabolic diseases [83,84]. These insights highlight the potential for personalized nutrition strategies to improve health outcomes by targeting specific dietary patterns and their associated BFIs.

### Limitations and Suggestions for Future Developments

This study’s strengths lie in its multidisciplinary nature, incorporating expertise from medical doctors, dietitians, laboratory technicians, and researchers. Additionally, this study’s focus on both short-term and long-term dietary patterns, as well as adherence to specific dietary indices like those related to the Mediterranean diet, provides a nuanced understanding of dietary behaviors. Furthermore, the integration of biochemical markers with dietary data through sophisticated statistical analyses and computational algorithms showcases this study’s innovative approach. The use of data training–testing ML demonstrates a rigorous methodology for identifying the predictors of dietary patterns. The development of a computational algorithm for predicting dietary patterns based on biochemical and lifestyle factors represents a significant advancement in precision nutrition research dietary intake assessment.

In terms of limitations, our study acknowledges several factors that may have influenced the results. The relatively small sample size may limit the generalizability of the findings and introduce potential biases that could affect the outcomes. Moreover, the machine learning models employed, while powerful, may be subject to limitations such as overfitting and restricted generalizability to broader populations. Additionally, there are opportunities for improvement in the design of our models. Enhancing the models’ capabilities to include a broader range of variables and integrating feedback mechanisms could improve their predictive accuracy and adaptability to individual patient needs. Addressing these limitations will be crucial for advancing the field of precision nutrition and ensuring that dietary interventions are both effective and sustainable.

The implications of our findings extend beyond the scope of this study, highlighting the importance of utilizing computational algorithms to bridge the gap between research and clinical practice. By leveraging these models, they can better tailor dietary recommendations to individual patients, ultimately promoting better health outcomes. This research underscores the need for the continued exploration of computational approaches in nutrition science, paving the way for future studies that can refine these algorithms and expand their application across diverse populations and clinical scenarios.

## 5. Conclusions

This pioneering pilot study determines that food intake can be characterized by phenotypical biomarkers, particularly distinguishing between the pro-Mediterranean and pro-Western clusters, indicating specific food consumption within the studied population. Moreover, the significant association between biological biomarkers, such as lipid profiles and other metabolic indicators, and dietary patterns underscores the relevance of these measurements in characterizing physiological responses to different diets. This research enforces the construction and validation of a computational algorithm for screening dietary patterns, where classification demonstrates the feasibility of using ML approaches in identifying and classifying eating habits, providing a potential tool for precision nutrition. Despite being a prototype, the results suggest that the integration of clinical phenotypical biomarkers and computational approaches may have clinical applications, offering valuable insights for the design of personalized nutritional interventions. It is crucial to emphasize that, given the pilot nature of this study, these conclusions should be considered seminal, encouraging further extensive and specific research to validate and expand upon these findings from precision nutrition and account for the need for consumption. To sum up, this investigation proved that integrating phenotypical and biochemical data enabled an accurate and valuable assessment of dietary pattern adherence based on computational algorithms.

## Figures and Tables

**Table 1:** Baseline characterization by sex of population.

	Male (*n* = 42)				Female (*n* = 96)				
	Mean	SD	P50	IQR	Mean	SD	P50	IQR	*p* ^1^
Age (years)	41.8	(14.3)	30.0	(43.5–53.0)	43.5	(14.2)	30.0	(42.5–55.0)	0.585
BMI (kg/m^2^)	28.3	(5.9)	24.4	(27.1–31.8)	26.2	(6.6)	21.3	(24.5–29.2)	0.024
Fat mass (%)	27.8	(9.3)	19.1	(29.0–35.1)	36.2	(9.9)	28.1	(35.5–43.7)	<0.001
Glucose (mg/dL)	102.9	(22.3)	89.0	(96.0–103.0)	93.1	(11.1)	85.5	(90.0–98.0)	0.015
Total cholesterol (mg/dL)	186.7	(37.1)	161.0	(182.5–207.0)	189.9	(35.4)	162.5	(186.0–214.5)	0.557
Triglycerides (mg/dL)	113.1	(108.1)	67.0	(89.0–116.0)	86.3	(51.2)	53.0	(74.0–110.0)	0.055
HDL-c (mg/dL)	53.6	(11.2)	51.5	(45.0–61.0)	64.9	(15.2)	63.5	(54.0–75.0)	<0.001
Physical dimension SF36 (0–100)	41.1	(7.5)	38.4	(42.8–46.4)	40.6	(6.4)	36.5	(42.8–45.4)	0.459
Mental dimension SF36 (0–100)	33.6	(6.2)	29.5	(33.6–38.3)	31.0	(7.1)	25.1	(31.6–36.8)	0.068
Total PA (h/week)	10.9	(8.6)	8.75	(5.3–14.3)	11.2	(8.4)	8.6	(5.7–14.5)	0.929
Vegetables (FFQ in g/d)	244.4	137.6	143.6	(227.9–323.8)	278.4	144.4	170.6	(245.8–373)	0.214
Fruits (FFQ in g/d)	200.5	145.9	82.6	(161.4–267.1)	313.2	349.9	132.9	(265.1–384.6)	0.030
Legumes (FFQ in g/d)	25.8	19.4	16.0	(17.1–34.3)	21.7	13.8	12.0	(18.8–27.7)	0.364
Cereals + potato (FFQ in g/d)	206.5	127.9	120.7	(191.5–235.7)	161.6	86.4	90.8	(162.5–223.7)	0.056
Dairy products (FFQ in g/d)	379.4	225.5	228.3	(317.3–567.4)	389.1	222.9	216.0	(370.5–553.6)	0.755
Olive oil (FFQ in g/d)	38.0	21.5	25.0	(35.0–50.0)	38.0	18.2	25.0	(35.0–50.0)	0.822
Fish (FFQ in g/d)	79.8	45.6	44.1	(79.7–105.7)	89.8	51.4	57.9	(78.6–117.2)	0.395
Nuts (FFQ in g/d)	25.0	23.0	7.1	(21.4–39.3)	26.1	25.8	7.1	(21.4–50.0)	0.904
Cookies, pastries, and sweets (FFQ in g/d)	50.6	51.2	12.9	(30.0–66.2)	34.9	33.5	8.7	(25.6–51.3)	0.103
White meat (FFQ in g/d)	67.6	28.7	64.3	(64.3–74.3)	63.6	48.9	21.4	(64.3–74.3)	0.086
Red meat (FFQ in g/d)	133.1	67.4	85.7	(133.5–167.3)	93.2	53.4	61.3	(82.6–123.5)	<0.001
Alcohol (FFQ in g/d)	6.9	8.2	1.4	(3.5–9.6)	2.8	3.2	0.0	(1.5–4.4)	0.003
	*n*	%			*n*	%			*p*
Smoking habit (%)									0.037
Non-smoker	19.0	(45.2)			63.0	(65.6)			
Former	11.0	(26.2)			21.0	(21.9)			
Smoker	12.0	(28.6)			12.0	(12.5)			
Number of prevalent diseases (%) ^2^									0.685
0	18	(42.8)			43	(35.4)			
1	12	(28.6)			33	(34.4)			
+2	12	(28.6)			29	(30.2)			

Abbreviations: BMI, body mass index; HDL, high-density lipoprotein; PA, physical activity. ^1^ *p*-value was calculated using Mann–Whitney U test or Chi2. ^2^ Cardiovascular disease, high blood pressure, hypertension, dyslipidemia, type 2 diabetes, obesity, depression, and insomnia.

**Table 2:** Cluster-stratified descriptive data: age- and sex-adjusted ^1^ 72 h dietary intake.

	Total (*n* = 138)			C1: Pro-MP (*n* = 86)			C2: Pro-WP (*n* = 52)			
	Non-C	Consumers		Non-C	Consumers		Non-C	Consumers		
	%	P50	(IQR)	%	P50	(IQR)	%	P50	(IQR)	*p* ^2^
Age		43.0	(30.0–55.0)		43.5	(30.0–55.0)		41.0	(29.5–51.5)	0.494
BMI		25.5	(21.9–30.5)		25.0	(21.4–30.4)		26.3	(22.1–31.3)	0.327
Vegetable group (g/d)	0.0%	43.3	(32.4–56.7)	0.0%	45.2	(33.5–56.6)	0.0%	41.6	(30.9–59.4)	0.507
Root tubers	8.7%	60.3	(30.5–86.4)	2.3%	61.2	(32.8–87.7)	19.2%	56.6	(23.6–80.0)	0.052
Pulse group (g/d)	52.2%	50.0	(30.0–100)	46.5%	45.0	(25.0–100)	61.5%	62.5	(42.1–140)	0.287
Canned	92.8%	150	(100–200)	95.3%	150	(15.0–200)	88.5%	150	(100–200)	0.130
Dried	64.5%	45.0	(25.0–77.1)	58.1%	45.0	(22.5–75.0)	75.0%	45.0	(41.4–77.1)	0.091
Cereal group (g/d)	1.4%	56.4	(38.0–76.3)	1.2%	52.2	(34.8–77.0)	1.9%	58.2	(44.7–71.2)	0.076
Breakfast	75.4%	35.9	(20.8–41.0)	82.6%	35.9	(20.0–43.3)	63.5%	40.0	(27.0–41.0)	0.010
Grain flour	18.8%	30.0	(12.4–54.7)	20.9%	30.0	(10.6–54.2)	15.4%	31.3	(14.0–57.5)	0.197
Drink group (g/d)	0.0%	403.1	(273–667)	0.0%	304	(230–383)	0.0%	751	(620–888)	<0.001
Alcoholic	40.6%	183	(56.7–330)	33.7%	200	(100–330)	51.9%	128	(25.4–330)	0.023
Non-alcoholic	0.0%	780	(416–1243)	0.0%	468	(309–733)	0.0%	1326	(996–1686)	<0.001
Commercial juices	90.6%	167	(50.0–200)	86.0%	100	(50.0–200)	98.1%	300	(300–300)	0.022
Meat group (g/d)	2.9%	57.5	(42.3–80.6)	1.2%	56.4	(41.2–84.5)	5.8%	62.2	(48.8–79.7)	0.489
Sausages	10.9%	34.5	(22.7–49.6)	11.6%	31.2	(20.8–48.3)	9.6%	40.0	(25.8–50.0)	0.084
Dairy product group (g/d)	0.7%	93.7	(70.9–131.3)	1.2%	99.9	(69.4–135)	0.0%	85.0	(71.6–125)	0.523
Milk	11.6%	106	(50.0–181.4)	10.5%	119	(58.2–200)	13.5%	99.2	(40.0–151.7)	0.163
Desserts	81.9%	96.7	(50.0–120)	86.0%	75.0	(40.0–125)	75.0%	100	(80.0–120)	0.091
Cheeses	9.4%	34.5	(20.0–75)	11.6%	30.0	(19.5–53.3)	5.8%	35.5	(24.7–102)	0.044
Fish group (g/d)	15.2%	64.0	(36.7–97.0)	14.0%	69.3	(37.2–102)	17.3%	62.0	(35.7–86.0)	0.172
Fatty	73.9%	88.8	(56.7–119)	79.1%	94.8	(62.4–148)	65.4%	68.9	(23.7–119)	0.127
Smoked	89.9%	50.0	(30.0–53.0)	86.0%	50.0	(30.0–53.0)	96.2%	5.0	(5.0–60.0)	0.056
Sweet group (g/d)	17.4%	16.0	(8.3–26.7)	16.3%	16.4	(8.7–28.8)	19.2%	15.0	(7.5–25.6)	0.330
Chocolates	47.8%	15.0	(10.0–28.0)	53.5%	15.0	(10.0–25.0)	38.5%	15.0	(10.0–30.0)	0.120
Industrial cakes	85.5%	50.0	(30.0–100)	82.6%	50.0	(22.5–140)	90.4%	50.0	(30.0–70.0)	0.196

Abbreviations: BMI, body mass index; C, cluster; IQR, interquartile range; Non-C, non-consumers; P, Percentile; pro-MP; pro-Mediterranean pattern; pro-WP, pro-Western pattern. ^1^ Data were adjusted for age and sex by inverse probability weighting method. ^2^ *p* was calculated using Mann–Whitney U test.

**Table 3:** Age- and sex-adjusted ^1^ descriptive data of baseline biochemical markers by cluster.

		Total (*n* = 138)		C1: Pro-MP (*n* = 86)		C2: Pro-WP (*n* = 52)		
	Normal Values	P50	(IQR)	P50	(IQR)	P50	(IQR)	*p* ^2^
Hematology								
White blood cells (10^3^/µL)	4.0–11.0	5.7	(4.9–6.8)	5.6	(4.9–6.5)	6.0	(5.1–7.2)	0.140
Lymphocytes (10^3^/µL)	1.0–4.8	1.8	(1.5–2.2)	1.8	(1.5–2.1)	1.9	(1.6–2.3)	0.096
Monocytes (10^3^/µL)	0.2–0.95	0.4	(0.3–0.5)	0.4	(0.3–0.5)	0.5	(0.4–0.5)	0.090
Basophils (10^3^/µL)	0.0–0.2	0.0	(0.0–0.1)	0.0	(0.0–0.1)	0.0	(0.0–0.1)	0.080
Coagulation/Blotting								
Activated Partial Thromboplastin Time (s)	25.0–35.0	31.0	(29.7–33.0)	30.8	(29.3–33.0)	31.2	(29.9–32.8)	0.153
Activated Partial Thromboplastin Time (ratio)	NA	1.0	(1.0–1.1)	1.0	(1.0–1.1)	1.1	(1.0–1.1)	0.166
General Biochemistry								
Total cholesterol (mg/dL))	<200	185	(162–214)	186	(164–222)	178	(160–208)	0.128
HDL cholesterol (mg/dL)	>40.0	60.0	(51.0–71.0)	63.0	(52.0–75.0)	55.0	(47.0–66.0)	0.004
Glomerular filtration rate CKD-EPI (mL/min/1.73 m^2^)	>60.0	91.0	(91.0–91.0)	91.0	(88.0–91.0)	91.0	(91.0–91.0)	0.041
Nutrition and Metabolism								
Zinc (µg/dL)	70.0–150	118	(109–129)	116	(108–126)	120	(112–132)	0.051
Ceruloplasmin (mg/dL)	20.0–60.0	26.5	(24.0–30.0)	26.5	(25.0–30.0)	25.0	(24.0–30.0)	0.138
Nutrition and metabolism								
Tocopherol/Cholesterol (ratio)	NA	7.5	(7.0–8.0)	7.4	(6.9–7.8)	7.6	(7.5–8.0)	0.031
Vitamin C (mg/L)	NA	10.3	(8.0–12.5)	10.3	(8.3–12.9)	10.3	(7.5–11.9)	0.056
Transferrin Saturation (%)	20.0–50.0	24.0	(18.0–32.0)	24.0	(18.0–33.0)	24.0	(18.0–28.0)	0.198

Abbreviations: C, cluster; CKD-EPI, Chronic Kidney Disease Epidemiology Collaboration; IQR, interquartile range; NA, Not Available; P, Percentile; pro-MP; pro-Mediterranean pattern; pro-WP, pro-Western pattern. ^1^ Data were adjusted for age and sex by inverse probability weighting method. ^2^ *p* was calculated using Mann–Whitney U test.

## Data Availability

The authors commit to making the data and materials associated with this research project readily available. Prior to requesting access, interested parties are encouraged to contact the PI of the project J. Alfredo Martínez (jalfredo.martinez@imdea.org) for information on data availability. Requests for data should be directed to the responsible PI, and access will be granted in accordance with ethical and legal considerations governing data sharing.

## Supplementary Materials

The following supporting information can be downloaded at https://www.mdpi.com/article/10.3390/nu16223817/s1, Figure S1: Determination of optimal number of clusters using elbow method (A) and silhouette (B) with bootstrapping, and (C) Dendrogram plot using ward.D2 distance; Figure S2. Cross-validation of elastic net regression model and selection of most important variables; Table S1: Cluster-stratified descriptive data: age- and sex-adjusted 72 h dietary intake in Dietary Deal study (n = 138); Table S2. Age- and sex-adjusted descriptive data of baseline biochemical parameters stratified by cluster in Dietary Deal study (n = 138); Table S3. Logistic Regression coefficients and 95% CI for probability of classification in pro-WD (C2) for selected variables; Table S4. Computational algorithm metrics; Table S5. Computational algorithm equations.
