# Supplementary material for: Translational Algorithms for Technological Dietary Quality Assessment Integrating Nutrimetabolic Data with Machine Learning Methods
Source: Nutrients. 2024 Nov 7;16(22):3817. doi: 10.3390/nu16223817 (PMC11597732; doi:10.3390/nu16223817)
Supplement: Supplementary file 1 [file nutrients-16-03817-s001.zip › nutrients-3263986-supplementary.pdf]

Figure S1. Determination of the optimal number of clusters using the elbow method (A) and silhouette (B) with bootstrapping, and (C) Dendrogram plot using ward.D2 distance.

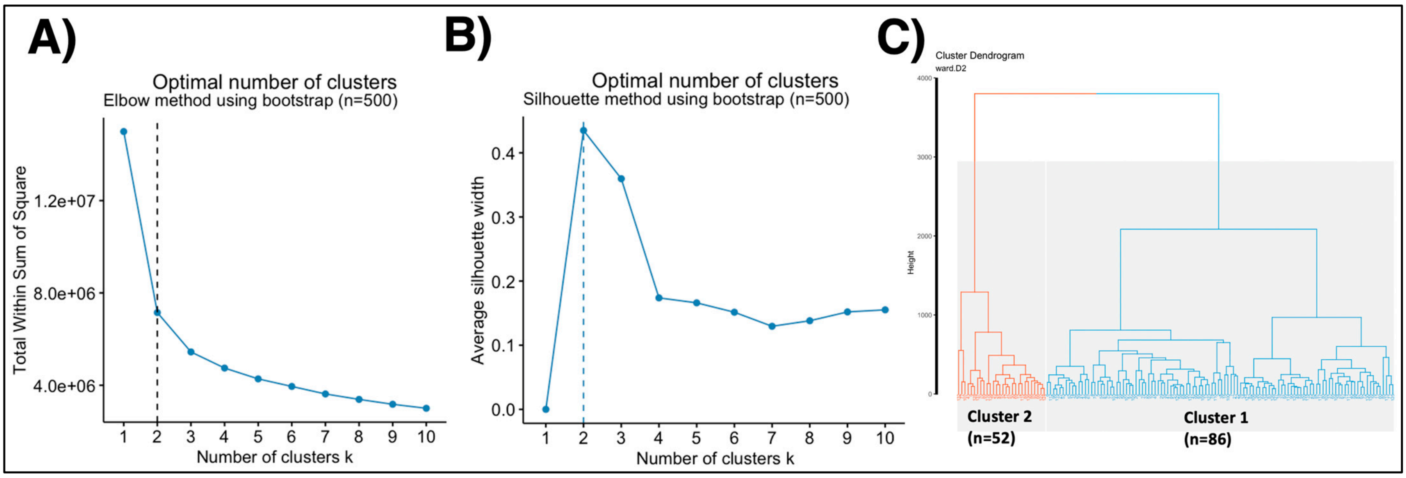

Table S1. Cluster-stratified descriptive data: age- and sex-adjusted 72-hour dietary intake in Dietary Deal study (n=138).

	Total (n=138)			C1: Pro-MD (n=86)			C2: Pro-WD (n=52)			
	Non-C	Consumers		Non-C	Consumers		Non-C	Consumers		
	%	p50	(IQR)	%	p50	(IQR)	%	p50	(IQR)	p[1]
**Age**		43	(30-55)		43.5	(30-55)		41	(29.5-51.5)	0.494
**BMI**		25.5	(21.9-30.5)		25	(21.4-30.4)		26.3	(22.1-31.3)	0.327
**Vegetables + fruits (g/d)**	0.0%	100.5	(69.6-136.7)	0.0%	95.7	(68.1-136.7)	0.0%	108.6	(79.7-136.2)	0.455
**Vegetables group (g/d)**	0.0%	43.3	(32.4-56.7)	0.0%	45.2	(33.5-56.6)	0.0%	41.6	(30.9-59.4)	0.507
Algae	98.6%	60	(0.6-60)	98.8%	60	(60-60)	98.1%	0.6	(0.6-0.6)	0.726
Fresh mushrooms	71.0%	32	(16-60)	72.1%	40	(16-61)	69.2%	32	(22-40)	0.811
Root tubers	8.7%	60.3	(30.5-86.4)	2.3%	61.2	(32.8-87.7)	19.2%	56.6	(23.6-80)	0.052
Frozen	81.2%	17.5	(7.5-66.7)	84.9%	21.5	(15-75)	75.0%	12.5	(7.5-33.8)	0.208
Canned	35.5%	20	(13-50)	38.4%	20	(12-52.5)	30.8%	20	(14-50)	0.471
Fresh	0.7%	39.1	(26.9-55.1)	1.2%	40.2	(27.2-55.7)	0.0%	36.8	(26.9-49.1)	0.613
**Fruits group (g/d)**	6.5%	60.8	(36.1-86.3)	9.3%	58.1	(32.5-86.3)	1.9%	66.7	(42.7-89.2)	0.248
Derivates	77.5%	16	(10-30)	79.1%	15	(12-30)	75.0%	16	(10-20)	0.616
Fresh	10.9%	74.8	(49.1-105.6)	12.8%	72.2	(45-106)	7.7%	76.1	(52.8-102.5)	0.292
Nuts	47.1%	20	(10-30)	47.7%	18	(8.5-24)	46.2%	20	(12.6-32)	0.504
Dried	86.2%	10.9	(7-16.8)	83.7%	10.9	(8-16)	90.4%	8	(7-40)	0.276
Natural fruit juices	78.3%	78.3	(10-200)	76.7%	78.3	(5-200)	80.8%	100	(10-200)	0.600
**Pulses group (g/d)**	52.2%	50	(30-100)	46.5%	45	(25-100)	61.5%	62.5	(42.1-140)	0.287
Derivates	90.6%	30	(25-50)	88.4%	34.4	(25-100)	94.2%	30	(18.8-34.9)	0.242
Canned	92.8%	150	(100-200)	95.3%	150	(15-200)	88.5%	150	(100-200)	0.130
Dried	64.5%	45	(25-77.1)	58.1%	45	(22.5-75)	75.0%	45	(41.4-77.1)	0.091
**Cereals group (g/d)**	1.4%	56.4	(38-76.3)	1.2%	52.2	(34.8-77)	1.9%	58.2	(44.7-71.2)	0.076
Breakfast	75.4%	35.9	(20.8-41)	82.6%	35.9	(20-43.3)	63.5%	40	(27-41)	0.010
Cookies	68.8%	30	(23.3-50)	68.6%	30	(20-47.3)	69.2%	30	(28-50)	0.835
Grains flour	18.8%	30	(12.4-54.7)	20.9%	30	(10.6-54.2)	15.4%	31.3	(14-57.5)	0.197
Bread	2.9%	58.8	(41.3-82)	2.3%	58.8	(37-80)	3.8%	59.3	(44.3-92.5)	0.358
Pasta	44.9%	80	(25-110)	44.2%	80	(28.8-112.5)	46.2%	70	(25-100)	0.745
**Oils and fats group (g/d)**	0.0%	14.3	(10.5-19.3)	0.0%	14.2	(10.5-18.3)	0.0%	14.4	(10.8-21.3)	0.443
Oils	0.0%	15	(11.2-21.4)	0.0%	14.6	(11.2-20.8)	0.0%	16.1	(11.5-23.1)	0.356
Margarine/Butters	51.4%	6.6	(4.1-10)	51.2%	6	(5-10)	51.9%	6.9	(4-10)	0.938
Other	83.3%	9.4	(1.7-15)	81.4%	9.4	(1.3-9.4)	86.5%	9.4	(6.6-25.8)	0.505
**Drinks group (g/d)**	0.0%	403.1	(273.3-666.8)	0.0%	304.1	(230.4-383.3)	0.0%	751.3	(620.2-888.6)	<0.001
Alcoholic	40.6%	182.5	(56.7-330)	33.7%	200	(100-330)	51.9%	128.3	(25.4-330)	0.023
Non-alcoholic	0.0%	779.9	(416.1-1243.8)	0.0%	467.7	(309.2-733.3)	0.0%	1326.3	(996.7-1686.3)	<0.001
Other	92.8%	250	(166.7-330)	93.0%	280	(100-330)	92.3%	250	(186.7-250)	0.853
Infusion/Tee/Coffees	8.7%	68.3	(40-116.7)	4.7%	82.9	(42-125)	15.4%	50	(30-79.3)	0.004
Commercial juices	90.6%	166.7	(50-200)	86.0%	100	(50-200)	98.1%	300	(300-300)	0.022
**Eggs (g/d)**	15.2%	42.6	(25.7-69.2)	18.6%	40.3	(26.2-57.7)	9.6%	52.2	(21.5-70.5)	0.203
**Meats group (g/d)**	2.9%	57.5	(42.3-80.6)	1.2%	56.4	(41.2-84.5)	5.8%	62.2	(48.8-79.7)	0.489
Poultry	34.1%	85.6	(42.7-128)	31.4%	74.3	(42.5-135)	38.5%	90	(51.2-120)	0.424
Pork	39.9%	60.2	(25.8-100)	37.2%	53.8	(25.8-90)	44.2%	71.1	(25.8-110)	0.904
Lamb	97.8%	77	(11.6-193)	97.7%	193	(11.6-193)	98.1%	77	(77-77)	0.876
Sausages	10.9%	34.5	(22.7-49.6)	11.6%	31.2	(20.8-48.3)	9.6%	40	(25.8-50)	0.084
Veal	43.5%	80	(40-119.2)	39.5%	81.5	(40-100)	50.0%	80	(51-125)	0.512
Viscera	94.2%	25	(16.7-120)	94.2%	25	(24.3-120)	94.2%	80	(16.7-125)	0.996
Other	94.9%	77.5	(60-105)	94.2%	82.7	(74.4-105)	96.2%	60	(60-77.5)	0.595
**Dairy products group (g/d)**	0.7%	93.7	(70.9-131.3)	1.2%	99.9	(69.4-135.7)	0.0%	85	(71.6-125.1)	0.523
Imitations	91.3%	180	(150-250)	93.0%	176.7	(150-180)	88.5%	226.7	(150-390)	0.329
Milk	11.6%	106.3	(50-181.4)	10.5%	118.8	(58.2-200)	13.5%	99.2	(40-151.7)	0.163
Creams	90.6%	27.5	(12.5-40)	90.7%	30	(12.5-40)	90.4%	27.5	(26.3-30)	0.938
Desserts	81.9%	96.7	(50-120)	86.0%	75	(40-125)	75.0%	100	(80-120)	0.091
Cheeses	9.4%	34.5	(20-75)	11.6%	30	(19.5-53.3)	5.8%	35.5	(24.7-102.2)	0.044
Yogurts	36.2%	125	(113-125)	39.5%	125	(112.5-125)	30.8%	125	(120-125)	0.327
**Fish group (g/d)**	15.2%	64	(36.7-97)	14.0%	69.3	(37.2-102.5)	17.3%	62	(35.7-86)	0.172
Derived canned	94.9%	50	(30-50)	94.2%	40	(30-50)	96.2%	50	(50-50)	0.627
Derived	58.7%	20	(10-67.5)	60.5%	20	(8.1-67.5)	55.8%	19.8	(15.3-87.5)	0.502
Fatty	73.9%	88.8	(56.7-119)	79.1%	94.8	(62.4-148)	65.4%	68.9	(23.7-119)	0.127
Lean	71.0%	102	(67.2-143)	69.8%	102	(53.4-143)	73.1%	112	(82.5-154)	0.719
Smoked	89.9%	50	(30-53)	86.0%	50	(30-53)	96.2%	5	(5-60)	0.056
Canned	55.8%	60	(30-80)	58.1%	70	(30-90)	51.9%	52	(20-77)	0.852
Other	93.5%	30	(30-35)	93.0%	30	(30-35)	94.2%	30	(20-45)	0.770
**Snacks (g/d)**	38.4%	24	(13.9-45)	38.4%	25	(12-40)	38.5%	20	(15.3-45)	0.719
**Sweet group (g/d)**	17.4%	16	(8.3-26.7)	16.3%	16.4	(8.7-28.8)	19.2%	15	(7.5-25.6)	0.330
Sugars	40.6%	8	(5.5-12.7)	39.5%	8.5	(6.3-12.7)	42.3%	7.3	(5-13.1)	0.384
Pastries	62.3%	50	(40-70)	62.8%	50	(50-62.5)	61.5%	45	(30-90)	0.830
Chocolates	47.8%	15	(10-28)	53.5%	15	(10-25)	38.5%	15	(10-30)	0.120
Sweets	88.4%	21	(15-55)	87.2%	50	(15-60)	90.4%	20	(15-20)	0.519
Other	93.5%	15	(10-30)	94.2%	10	(10-22.5)	92.3%	30	(15-45)	0.644
Industrial cakes	85.5%	50	(30-100)	82.6%	50	(22.5-140)	90.4%	50	(30-70)	0.196
**Proccesed food group (g/d)**	35.5%	5	(1.4-56.7)	34.9%	5	(1.4-50.4)	36.5%	30	(1.8-80.3)	0.523
**Condiments (g/d)**	0.0%	1.5	(0.8-2.8)	0.0%	1.6	(0.8-2.8)	0.0%	1.4	(0.9-3)	1.000
**Sauces (g/d)**	44.9%	15	(10-23.8)	46.5%	20	(10-25)	42.3%	15	(8.3-20)	0.956

Abbreviations: BMI, Body Mass Index; C, cluster; IQR, Interquartile Range; Non-C, Non-Consumers; P, Percentile; Pro-MP; Pro-Mediterranean Pattern; Pro-WP, Pro-Western Pattern. All data were adjusted for age and sex using de inverse probability weighting. [1] p was calculated using the U Mann-Whitney test.

Table S2. Age- and sex-adjusted descriptive data of baseline biochemical parameters stratified by cluster in Dietary Deal study (n=138).

		Total (n=138)		C1: Pro-MD (n=86)		C2: Pro-WD (n=52)		
	Normal values	p50	IQR	p50	IQR	p50	IQR	p[1]
**Hematology**								
Red blood cells (10^6/µl)	4.5-6.0	4.7	(4.3-5)	4.7	(4.4-4.9)	4.7	(4.3-5)	0.531
Hemoglobin (g/dL)	13.8-15.1	14	(13.1-14.6)	13.9	(13.3-14.8)	14.1	(13-14.6)	0.622
Mean corpuscular volume (fl)	80-100	90.3	(87.7-92.4)	90.3	(87.9-92.7)	90.3	(87.6-92)	0.577
Mean corpuscular hemoglobin concentration (g/dL)	32-36	33.3	(33-33.8)	33.4	(33-33.8)	33.2	(33-33.8)	0.560
Red Cell Blood Distribution Width (%)	11.5-14.5	13.8	(13.2-14.2)	13.6	(13.1-14)	13.9	(13.3-14.3)	0.238
Red Cell Blood Distribution Width (SD, fL)	NA	43.8	(42-45.1)	43.3	(42-44.6)	44.2	(42-45.1)	0.536
Platelet Count (10^3/µl)	150-450	241	(208-277)	241	(209-274)	241	(206-302)	0.626
Mean Platelet Volume (fL)	7.4-10.4	9.1	(8.5-9.8)	9	(8.5-9.7)	9.2	(8.7-10.1)	0.245
White blood cells (10^3/µl)	4-11	5.7	(4.9-6.8)	5.6	(4.9-6.5)	6	(5.1-7.2)	0.140
Neutrophils (10^3/µl)	2.5-7	3.2	(2.6-4.2)	3.1	(2.5-4)	3.5	(2.8-4.4)	0.265
Lymphocytes (10^3/µl)	1-4,8	1.8	(1.5-2.2)	1.8	(1.5-2.1)	1.9	(1.6-2.3)	0.096
Monocytes (10^3/µl)	0.2-0.95	0.4	(0.3-0.5)	0.4	(0.3-0.5)	0.5	(0.4-0.5)	0.090
Eosinophils (10^3/µl)	0.15-0.5	0.1	(0.1-0.2)	0.1	(0.1-0.2)	0.1	(0.1-0.2)	0.463
Basophils (10^3/µl)	0-0.2	0	(0-0.1)	0	(0-0.1)	0	(0-0.1)	0.080
**Coagulation/Blotting**								
Prothrombin Time (s)	11-13.5	11.2	(10.7-11.6)	11.2	(10.7-11.5)	11.1	(10.7-11.7)	0.783
Activated Partial Thromboplastin Time (s)	25-35	31	(29.7-33)	30.8	(29.3-33)	31.2	(29.9-32.8)	0.153
Activated Partial Thromboplastin Time (ratio)	NA	1	(1-1.1)	1	(1-1.1)	1.1	(1-1.1)	0.166
PT-derived fibrinogen (mg/dL)	200-400	433	(380-509)	429	(379-514)	454	(383-509)	0.714
**General Biochemiestry**								
Glucose (mg/dL)	70-100	93	(86-100)	94	(86-101)	93	(86-98)	0.583
Bilirubin (mg/dL)	0.1-1.2	0.6	(0.5-0.8)	0.6	(0.5-0.8)	0.6	(0.4-0.8)	0.802
Total cholesterol (mg/dL))	<200	185	(162-214)	186	(164-222)	178	(160-208)	0.128
HDL cholesterol (mg/dL)	>40	60	(51-71)	63	(52-75)	55	(47-66)	0.004
Triglycerides (mg/dL)	<150	76	(57-110)	76	(59-107)	78	(52-117)	0.601
Creatinine (mg/dL)	0.6-1.3	0.8	(0.7-0.9)	0.8	(0.7-0.9)	0.8	(0.7-0.9)	0.679
CKD-EPI (mL/min/1.73m2)	>60	91	(91-91)	91	(88-91)	91	(91-91)	0.041
Urea (mg/dL)	10-50	33	(29-37)	33	(29-37)	33	(29-38)	0.911
Uric acid (mg/dL)	3.4-6	4.9	(4.1-5.6)	4.9	(4.1-5.6)	4.9	(4.2-5.6)	0.372
Total proteins (g/dL)	6.0-8.3	7.3	(7.1-7.6)	7.3	(7.1-7.5)	7.4	(7-7.6)	0.540
Albumin (g/dL)	3.4-5.4	4.4	(4.2-4.6)	4.4	(4.2-4.6)	4.3	(4.2-4.5)	0.690
**Liver function**								
Alanine aminotransferase (U/L)	7-56	19	(14-25)	19	(14-25)	19	(14-26)	0.255
Aspartate aminotransferase (U/L)	10-40	21	(18-25)	21	(18-25)	21	(19-25)	0.744
Alkaline phosphatase (U/L)	30-120	68	(55-83)	68	(55-83)	68	(57-82)	0.527
Gamma glutamyl transferase (U/L)	9-48	19	(14-27)	20	(14-28)	18	(14-25)	0.585
**Minerals and electrolytes**								
Calcium (mg/dL)	8.5-10.5	9.8	(9.6-10)	9.8	(9.5-10)	9.8	(9.6-10)	0.975
Phosphorus (mg/dL)	2.5-4.5	3.5	(3.2-3.8)	3.5	(3.2-3.9)	3.4	(3.1-3.7)	0.600
Magnesium (mg/dL)	1.6-2.6	2	(2-2.1)	2	(2-2.1)	2	(2-2.1)	0.798
Sodium (mmol/L)	135-145	138	(137-140)	138	(137-140)	138	(137-139)	0.271
Potassium (mmol/L)	3.5-5.1	4.2	(4.1-4.4)	4.2	(4-4.4)	4.2	(4.1-4.4)	0.966
Chlorine (mmol/L)	95-105	104	(103-105)	104	(103-106)	104	(103-105)	0.303
**Inflammation**								
C-reactive protein (mg/L)	<1	1	(0.5-2.8)	1	(0.5-2.8)	0.9	(0.6-2.6)	0.961
Interleukin-6 (pg/mL)	NA	1.5	(1.4-2.7)	1.5	(1.4-3)	1.4	(1.4-2.7)	0.438
**Nutrition and Metabolism**								
Copper (µg/dL)	70 - 140	115	(104-132)	115	(105-132)	115	(104-126)	0.432
Zinc (µg/dL)	70 - 150	118	(109-129)	116	(108-126)	120	(112-132)	0.051
Transthyretin prealbumin (mg/dL)	16 - 40	25	(23-28)	25	(23-28)	26	(22-28)	0.483
Retinol-binding protein (mg/dL)	2.1 - 6.1	4.7	(4-5.6)	4.7	(4-5.7)	4.9	(4-5.6)	0.684
Ceruloplasmin (mg/dL)	20 - 60	26.5	(24-30)	26.5	(25-30)	25	(24-30)	0.138
**Hormones**								
TSH (µUI/mL)	0.4 - 4.0	1.8	(1.3-2.3)	1.8	(1.3-2.5)	1.8	(1.3-2.2)	0.918
Free T4 (pg/mL)	0.8 - 2.0	8.8	(8-9.8)	8.9	(8-10.1)	8.8	(8-9.8)	0.504
Insulin (µUI/mL)	NA	8.4	(4.4-13.5)	8.3	(4.6-13.5)	9.8	(3.9-13.7)	0.800
Intact parathyroid hormone (pg/mL)	10 - 65	47.7	(38-58.5)	47.7	(37.1-59.6)	47.4	(38.4-56.8)	0.402
**Nutrition and metabolism**								
Homocysteine (µmol/L)	<15	11	(9-13)	11	(9-13)	10	(9-13)	0.484
Retinol (mg/L)	NA	0.6	(0.5-0.7)	0.6	(0.5-0.7)	0.6	(0.5-0.7)	0.491
Tocopherol (mg/L)	NA	14	(12.3-16.1)	14	(12.3-16.2)	14	(12.6-15)	0.912
Tocopherol/Cholesterol (ratio)	NA	7.5	(7-8)	7.4	(6.9-7.8)	7.6	(7.5-8)	0.031
Vitamin C (mg/L)	NA	10.3	(8-12.5)	10.3	(8.3-12.9)	10.3	(7.5-11.9)	0.056
Vitamin D 25-OH (ng/mL)	30 - 100	23	(15-30)	23	(14-30)	23	(16-29)	0.906
Folate (ng/mL)	3 - 20	7.3	(5.2-9.9)	7.4	(5-9.9)	7.2	(5.8-9.2)	0.604
Cobalamin (pg/mL)	200 - 900	288	(215-389)	266	(220-396)	288	(212-375)	0.920
Total Serum Iron (µg/dL)	60 - 170	95	(70-115)	95	(70-120)	91	(70-109)	0.372
Ferritin (ng/mL)	24-307	52	(25-107)	58	(27-107)	44	(21-107)	0.493
Transferrin (mg/dL)	200 - 360	266.5	(247-294)	265	(243-293)	270	(248-299)	0.335
Transferrin Saturation (%)	20 - 50	24	(18-32)	24	(18-33)	24	(18-28)	0.198
HbA1c (%)	<5.7	5.4	(5.2-5.7)	5.4	(5.2-5.6)	5.4	(5.2-5.8)	0.545

Abbreviations: BMI, Body Mass Index; C, cluster; CKD-EPI, Chronic Kidney Disease Epidemiology Collaboration; HbA1c, Glycosylated Hemoglobin 1AC; IQR, Interquartile Range; NA, No Available; P, Percentile; Pro-MP; Pro-Mediterranean Pattern; Pro-WP, Pro-Western Pattern. All data were adjusted for age and sex using de inverse probability weighting. [1] p was calculated using the U Mann-Whitney test.

Figure S2. Cross-Validation of elastic net regression model and selection of most important variables

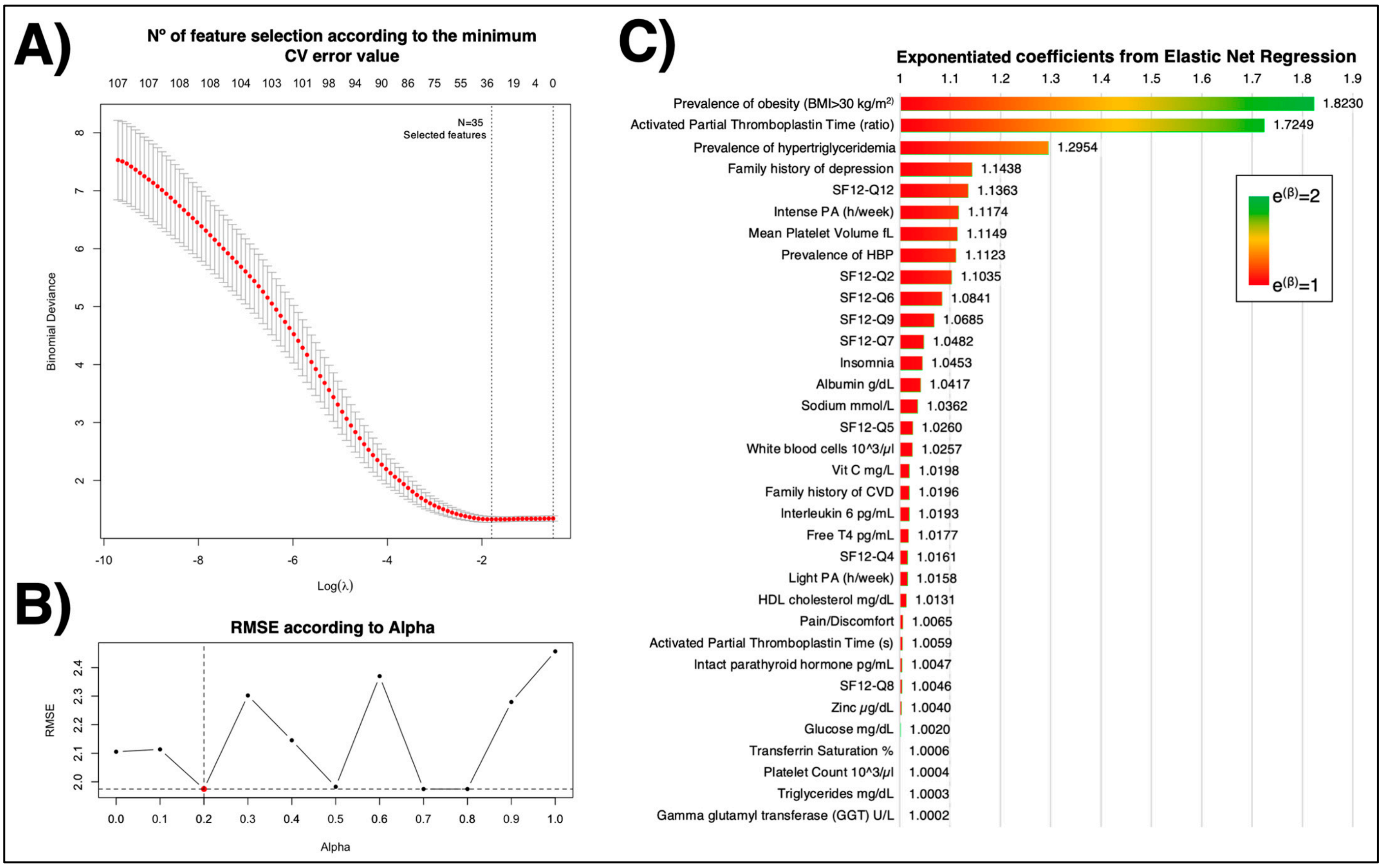

A) Prediction error estimation through cross-validation and 95% confidence interval: selection of the minimum cross-validation error value; B) Visualization of the optimal alpha value according to RMSE; C) Exponentiated coefficients of the selected variables in the elastic net regression model. Abbreviations: PA, physical activity, CVD, cardiovascular disease, HBP, high blood pressure, RMSE, Root Mean Square Error, CV, cross-validation.

Table S3. Logistic regression coefficients and 95% CI for probability of classification in Pro-WD (C2) for selected variables.

	Main model (R^2=81.9%)				Supervised model only biochemistry (R^2=16.16%)			
Variable	β	95% CI	p	ΔR[a]	β	95% CI	p	ΔR[a]
Intercept	+1664.327				19.7985			
Platelet Count (10^3/µl)	-0.0002	(-0.0494 to 0.0491)	0.995	0	0.0056	(-0.0027 to 0.0138)	0.186	-0.292
Mean Platelet Volume (fL)	+6.8231	(0.9546 to 12.6916)	0.023	-0.078	0.5411	(0.0333 to 1.0489)	0.037	-0.307
White blood cells (10^3/µl)	+2.1869	(-0.6996 to 5.0735)	0.138	-0.012	0.0756	(-0.1871 to 0.3383)	0.573	-0.283
Activated Partial Thromboplastin Time (s)	-21.7519	(-38.7764 to -4.7275)	0.012	-0.137	-	-	-	-
Activated Partial Thromboplastin Time (ratio)	+636.7128	(139.6156 to 1133.81)	0.012	-0.135	-	-	-	-
Glucose (mg/dL)	+0.4355	(-0.0088 to 0.8798)	0.055	-0.037	-0.0107	(-0.0409 to 0.0194)	0.485	-0.284
HDL cholesterol (mg/dL)	-0.4922	(-0.9858 to 0.0014)	0.051	-0.058	-0.0386	(-0.0714 to -0.0059)	0.021	-0.315
Albumin (g/dL)	-0.1996	(-0.3817 to -0.0174)	0.032	-0.078	-0.7340	(-2.2912 to 0.8232)	0.356	-0.286
Triglycerides (mg/dL)	+20.0017	(0.4076 to 39.5958)	0.045	-0.036	-0.0056	(-0.0137 to 0.0025)	0.178	-0.295
Gamma glutamyl transferase (U/L)	-0.1752	(-0.3252 to -0.0253)	0.022	-0.075	-0.0065	(-0.0277 to 0.0147)	0.546	-0.285
Sodium (mmol/L)	-12.5937	(-24.2484 to -0.9391)	0.034	-0.124	-0.1652	(-0.3921 to 0.0617)	0.154	-0.293
Interleukin-6 (pg/mL)	+1.5849	(-0.1081 to 3.2779)	0.067	-0.024	-	-	-	-
Zinc (µg/dL)	+0.8951	(0.0414 to 1.7488)	0.040	-0.059	0.0325	(0.0031 to 0.0620)	0.031	-0.308
Free T4 (pg/mL)	-5.0115	(-9.7372 to -0.2858)	0.038	-0.065	-	-	-	-
Vitamin C (mg/L)	-0.3195	(-1.6338 to 0.9948)	0.634	-0.001	-0.0871	(-0.2101 to 0.0359)	0.165	-0.292
Transferrin Saturation (%)	-0.4308	(-0.8519 to -0.0096)	0.045	-0.026	-0.0040	(-0.0520 to 0.0439)	0.869	-0.282
Intact parathyroid hormone (pg/mL)	+0.0281	(-0.1777 to 0.2339)	0.789	0	-	-	-	-
Intense PA (h/week)	+2.4947	(0.4787 to 4.5106)	0.015	-0.112	-	-	-	-
Light PA (h/week)	+2.0847	(0.2802 to 3.8891)	0.024	-0.121	-	-	-	-
Family history of CVD ("No" as ref.)	+19.9037	(-1.7891 to 41.5965)	0.072	-0.030	-	-	-	-
Family history of depression ("No" as ref.)	+33.0262	(4.5376 to 61.5147)	0.023	-0.063	-	-	-	-
Prevalence of hypertriglyceridemia ("No" as ref.)	+24.2007	(1.5521 to 46.8492)	0.036	-0.091	-	-	-	-
Prevalence of HBP ("No" as ref.)	+28.5392	(1.3187 to 55.7598)	0.040	-0.040	-	-	-	-
Prevalence of obesity ("No" as ref.)	+108.0937	(-4.0217 to 220.209)	0.059	-0.069	-	-	-	-
Prevalence of insomnia ("Never" as ref.)				-0.021				-
Seldom	-7.8722	(-17.96 to 2.2156)	0.126		-	-	-	
Yes (often or in the past)	-9.823	(-32.2055 to 12.5595)	0.390		-	-	-	
SF12-Q2 ("Yes, it limits me a lot" as ref.)				-0.058				-
Yes, it limits me a little	-98.8947	(-191.0768 to -6.7126)	0.035		-	-	-	
No, it doesn't limit me at all	-91.2861	(-177.0326 to -5.5396)	0.037		-	-	-	
SF12-Q4 ("No" as ref.)	+15.8967	(0.4116 to 31.3818)	0.044	-0.026	-	-	-	-
SF12-Q5 ("No" as ref.)	-21.9897	(-45.7357 to 1.7564)	0.070	-0.023	-	-	-	-
SF12-Q6 ("No" as ref.)	-63.5839	(-121.3566 to -5.8111)	0.031	-0.071	-	-	-	-
SF12-Q7 ("No" as ref.)	-7.5112	(-45.6845 to 30.6621)	0.700	-0.001	-	-	-	-
SF12-Q8 ("Not at all" as ref.)				-0.139				-
A little	-31.5814	(-59.5651 to -3.5978)	0.027		-	-	-	
Regular	+42.7036	(2.8897 to 82.5174)	0.036		-	-	-	
Quite a bit	-18.2459	(-45.4624 to 8.9705)	0.189		-	-	-	
A lot	+68.7056	(6.9647 to 130.4465)	0.029		-	-	-	
SF12-Q9 ("Always" as ref.)				-0.202				-
Almost always	+6.9868	(-7.3832 to 21.3567)	0.341		-	-	-	
Many times	-32.9719	(-63.8234 to -2.1203)	0.036		-	-	-	
Sometimes	+1.2236	(-15.7152 to 18.1623)	0.887		-	-	-	
Only sometimes	+47.0958	(-0.9011 to 95.0927)	0.054		-	-	-	
Never	-51.3751	(-110.4913 to 7.7411)	0.089		-	-	-	
SF12-Q12 ("Always" as ref.)				-0.166				-
Almost always	-56.4891	(-141.6452 to 28.667)	0.194		-	-	-	
Many times	+39.1117	(-21.4343 to 99.6577)	0.205		-	-	-	
Sometimes	-30.5748	(-95.1579 to 34.0084)	0.353		-	-	-	
Only sometimes	-30.2242	(-90.2551 to 29.8068)	0.324		-	-	-	
Never	-79.1236	(-176.2876 to 18.0405)	0.110		-	-	-	
Pain/Discomfort ("I have no pain or discomfort" as ref.)				-0.148				-
I have moderate pain or discomfort	-99.8898	(-187.871 to -11.9085)	0.026		-	-	-	
I have a lot of pain or discomfort	-169.752	(-317.0802 to -22.4238)	0.024		-	-	-	

Abbreviations: PA, physical activity, C, cluster, CI, confident interval, CVD, cardiovascular disease, HBP, high blood pressure

[a] ΔR represents the R change after subtracting each variable from full model.

Table S4. Computational algorithms metrics.

	Computational algorithm	
	**Main**	**Supervised only biochemistry**
**R^2 (%)**	81.86	16.16
**AUC**	99.22	76.05
**PR AUC**	58.64	60.24
**TP (Sensitivity, %)**	96.15	53.85
**TN (Specificity, %)**	97.67	82.56
**FP (%)**	3.85	34.88
**FN (%)**	2.33	25.26
**Correctly classified (%)**	97.10	71.74
**Positive predictive value (%)**	96.15	65.12
**Negative predictive value (%)**	97.67	74.74

Abbreviations: AUC, area under curve, Dif, differences, PR, precision-recall, TP, true positive, TN, true negative, FP, false positive, FN, false negative.

Table S5. Computational algorithms equations.

Algorithm	Classification Formula	Equation from Logistic Regression
Main	$\Pr(\text{cluster} = 2) = \frac{1}{1 + e^{-(\mathbf{xj\beta})}}$	**xjβ** = **1664.327** + (Platelet Count 10^3/µl × **-0.0002**) + (Mean Platelet Volume fL × **6.8231**) + (White blood cells 10^3/µl × **2.1869**) + (Activated Partial Thromboplastin Time s × **-21.7519**) + (Activated Partial Thromboplastin Time ratio × **636.7128**) + (Glucose mg/dL × **0.4355**) + (HDL cholesterol mg/dL × **-0.4922**) + (Triglycerides mg/dL × **-0.1996**) + (Albumin g/dL × **20.0017**) + (Gamma glutamyl transferase U/L × **-0.1752**) + (Sodium mmol/L × **-12.5937**) + (Interleukin 6 pg/mL × **1.5849**) + (Zinc µg/dL × **0.8951**) + (Free T4 pg/mL × **-5.0115**) + (Vit C mg/L × **-0.3195**) + (Transferrin Saturation % × **-0.4308**) + (Intact parathyroid hormone pg/mL × **0.0281**) + (Intense PA h/week × **2.4947**) + (Light PA h/week × **2.0847**) + (Family history of CVD × **β category**) + (Family history of depression × **β category**) + (Prevalence of hypertriglyceridemia × **β category**) + (Prevalence of HBP × **β category**) + (Prevalence of obesity × **β category**) + (Prevalence of insomnia × **β category**) + (SF12-Q2 × **β category**) + (SF12-Q4 × **β category**) + (SF12-Q5 × **β category**) + (SF12-Q6 × **β category**) + (SF12-Q7 × **β category**) + (SF12-Q8 × **β category**) + (SF12-Q9 × **β category**) + (SF12-Q12 × **β category**) + (Pain/Discomfort × **β category**)
Supervised	$\Pr(\text{cluster} = 2) = \frac{1}{1 + e^{-(\mathbf{xj\beta})}}$	**xjβ** = **60.8722** + (Platelet Count 10^3/µl × **0.0041**) + (Mean Platelet Volume fL × **0.7605**) + (White blood cells 10^3/µl × **0.114**) + (Glucose mg/dL × **-0.0309**) + (HDL cholesterol mg/dL × **-0.0476**) + (Albumin g/dL × **-0.2732**) + (Triglycerides mg/dL × **-0.0066**) + (Gamma glutamyl transferase U/L × **-0.0088**) + (Sodium mmol/L × **-0.4756**) + (Zinc µg/dL × **0.0501**) + (Vitamin C mg/L × **-0.1728**) + (Transferrin Saturation % × **-0.0035**) + (Intense PA h/week × **0.5309**) + (Light PA h/week × **0.1237**) + (Family history of CVD × **β category**) + (Prevalence of HBP × **β category**) + (Prevalence of obesity × **β category**) + (Prevalence of insomnia × **β category**) + (SF12-Q9 × **β category**) + (SF12-Q12 × **β category**)
Supervised only biochemistry	$\Pr(\text{cluster} = 2) = \frac{1}{1 + e^{-(\mathbf{xj\beta})}}$	**xjβ** = **19.7985** + (Platelet Count 10^3/µl × **0.0056**) + (Mean Platelet Volume fL × **0.5411**) + (White blood cells 10^3/µl × **0.0756**) + (Glucose mg/dL × **-0.0107**) + (HDL cholesterol mg/dL × **-0.0386**) + (Albumin g/dL × **-0.7340**) + (Triglycerides mg/dL × **-0.0056**) + (Gamma glutamyl transferase U/L × **-0.0065**) + (Sodium mmol/L × **-0.1652**) + (Zinc µg/dL × **0.0325**) + (Vitamin C mg/L × **-0.0871**) + (Transferrin Saturation % × **-0.0040**)

Abbreviations: APTT, Activated Partial Thromboplastin Time, MPV, Mean Platelet Volume, IL6, Interleukin-6, GGT, Gamma glutamyl transferase, PA, physical activity, CVD, cardiovascular disease, HBP, high blood pressure.
